# The relationship between C-reactive protein to lymphocyte ratio and the prevalence of chronic kidney disease in US adults: a cross-sectional study

**DOI:** 10.3389/fendo.2024.1469750

**Published:** 2025-01-15

**Authors:** Pengfei He, Jiao Zhang, Ni Tian, Yuanyuan Deng, Min Zhou, Cheng Tang, Yu Ma, Mianzhi Zhang

**Affiliations:** ^1^ Dongfang Hospital, Beijing University of Chinese Medicine, Beijing, China; ^2^ Department of Nephrology, Tianjin Academy of Traditional Chinese Medicine Affiliated Hospital, Tianjin, China; ^3^ Dongzhimen Hospital, Beijing University of Chinese Medicine, Beijing, China

**Keywords:** C-reactive protein, lymphocytes, chronic kidney disease, inflammation, National Health and Nutrition Examination Survey

## Abstract

**Objective:**

The C-reactive protein/Lymphocyte Ratio (CLR) is a novel biomarker whose role in the development of chronic kidney disease (CKD) is not well understood. This study aimed to investigate the correlation between CLR and the prevalence of CKD.

**Methods:**

This cross-sectional study included participants from the US National Health and Nutrition Examination Survey conducted between 1999 and 2010. Multivariate regression analyses and subgroup analyses were performed, controlling for socio-demographic variables, lifestyle behaviors, chronic diseases associated with kidney disease, and biochemical markers of bone metabolism. The associations between CLR and CKD prevalence, as well as indicators of renal damage, were explored. Non-linear relationships were analyzed using weighted restricted cubic splines. The predictive ability of CLR for CKD was assessed by the receiver operating characteristic curve and the area under the curve was calculated. Subgroup and sensitivity analyses were conducted to validate the robustness of the model.

**Results:**

A total of 13,862 respondents were included, comprising 2,449 CKD patients and 11,413 non-CKD patients. Weighted logistic regression modeling revealed a positive correlation between CLR levels and CKD prevalence (Odds ratio [OR] = 1.54, 95% Confidence interval [CI] = 1.30 to 1.83, P < 0.001). Additionally, CLR levels were negatively correlated with the glomerular filtration rate, a marker of renal injury, and positively correlated with the urinary albumin/creatinine ratio. The receiver operating characteristic curve demonstrated that the area under the curve for CLR in predicting CKD was 0.653 (95% CI, 0.641–0.665). The optimal cutoff value was 0.856, with a sensitivity of 0.703, specificity of 0.526, positive predictive value of 0.874, and negative predictive value of 0.275. The robustness of the model was confirmed through subgroup and sensitivity analyses.

**Conclusion:**

Analysis of a large cross-sectional dataset demonstrated a positive correlation between CLR levels and CKD prevalence, suggesting that CLR may serve as a novel marker for the development and treatment of CKD.

## 1 Introduction

Chronic kidney disease (CKD) is a serious public health concern and has become one of the leading causes of mortality worldwide (1). In recent years, the incidence of CKD has been steadily increasing, with studies showing that approximately 843.6 million individuals globally are affected (2), and the prevalence in the general population is as high as 14.3% (3). CKD is characterized by structural or functional abnormalities of the kidneys, defined by an estimated glomerular filtration rate (eGFR) of less than 60 ml/min/1.73 m² or the presence of markers of kidney damage for more than three months. Clinical presentations of CKD can range from asymptomatic to symptoms such as foamy urine, hematuria, decreased urine output, increased nocturia, nausea, fatigue, loss of appetite, and pruritus, often overlooked when symptoms are mild. The etiology of CKD is diverse, with diabetes, cardiovascular disease, hypertension, and obesity identified as significant risk factors (4). The pathogenesis of CKD involves various mechanisms, including inflammation, immune responses, and podocyte autophagy. Systemic chronic inflammation plays a pivotal role in the pathogenesis of CKD, driving research into the involvement of inflammatory markers in CKD progression, including C-reactive protein (CRP), interleukins, tumor necrosis factor, interferons, and chemokines (5–8). For instance, elevated IL-6 levels in CKD patients have been associated with a greater decline in eGFR (9), although Salimi et al. found no association between baseline IL-6 levels and eGFR in CKD patients (10). The systemic immune-inflammation index, which combines platelet, lymphocyte, and neutrophil counts in peripheral blood, has been shown to have a positive correlation with the incidence of CKD (11). Therefore, more precise inflammatory markers are being investigated for their role in CKD development.

The C-reactive protein/Lymphocyte Ratio (CLR) is a novel biomarker, calculated as the ratio of CRP to lymphocytes, reflecting systemic inflammation and immune response. An increase in CLR indicates heightened systemic inflammation and a decreased immune response (12, 13). Recent studies have demonstrated that CLR is associated with tumors, postoperative infections, COVID-19, acute pancreatitis, dilated cardiomyopathy, and myocardial infarction, highlighting its potential as an emerging inflammatory marker in a range of inflammatory and immune-related diseases (14–19). This study aimed to investigate the correlation between CLR and CKD incidence and indicators of kidney injury, providing insights into the development and prognosis of CKD.

## 2 Materials and methods

### 2.1 Study population

**Figure 1:**
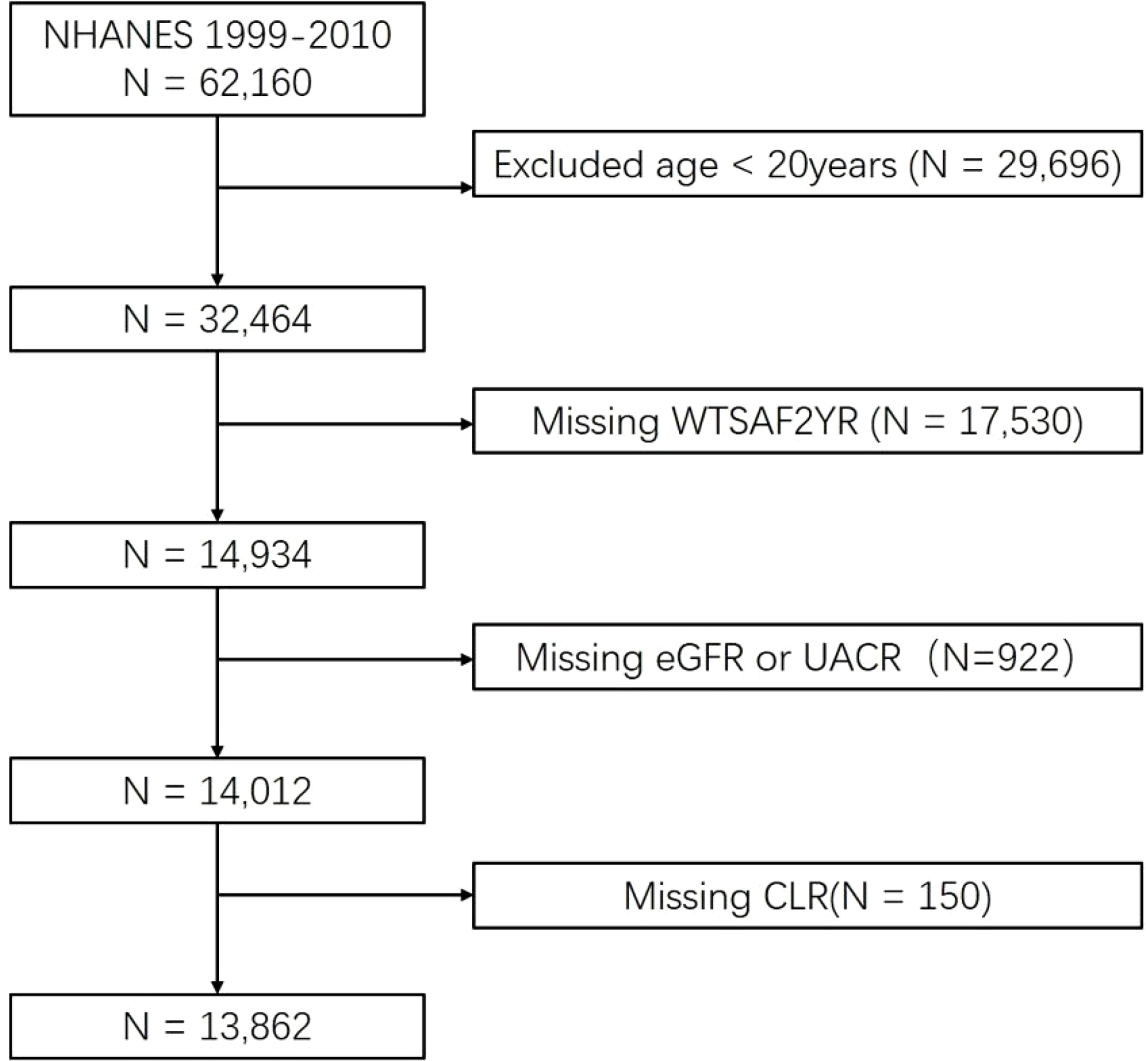
Flow chart of the study population inclusion. CLR, C-reactive protein to lymphocyte ratio; eGFR, estimated glomerular filtration rate; UACR, urinary albumin-to-creatinine ratio.

The National Health and Nutrition Examination Survey (NHANES) is a population-based, nationally representative cross-sectional survey (https://www.cdc.gov/nchs/nhanes/about_nhanes.htm) conducted by the US Centers for Disease Control and Prevention. Informed consent was obtained from each participant. Our study included data from six cycles: 1999-2000, 2001-2002, 2003-2004, 2005-2006, 2007-2008, and 2009-2010. Participants under 18 years of age were excluded due to the lack of education, poverty-to-income ratio (PIR), and chronic disease data. After further excluding individuals under 20 years of age, those with missing weighted values, missing CKD assessment indicators, eGFR and urinary albumin-to-creatinine ratio (UACR), and missing data on CLR, a total of 13,862 respondents from the NHANES database (1999-2010) were included in this study (**Figure 1**).

### 2.2 Assessment of CLR

CRP levels and peripheral blood lymphocyte counts were measured using automated blood analysis equipment. CLR was calculated as follows (20):[CRP level (mg/dL)×100]/peripheral blood lymphocyte count (pcs/μl).

### 2.3 Assessment of CKD

CKD is defined as fulfilling any of the following (21, 22): eGFR<60mL/min/1.73m^2^ (2); UACR ≥30mg/g. The eGFR was calculated based on the CKD epidemiological collaboration equation (23): 142×min(Scr/κ, 1)^α^×max(Scr/κ, 1)^-1.209^×0.993^Age^×1.012[if female], where κ = 0.7 (females) or 0.9 (males), a= -0.241(female) or 0.302(male), Scr = serum creatinine in mg/dL, The “min(Scr/κ, 1)” factor indicates the minimum of Scr/κ or 1.0 and “max(Scr/κ, 1)” indicates the maximum of Scr/κ or 1.0.

### 2.4 Covariates

Four categories of covariates were included in this study, including socio-demographic information (gender, age, ethnicity, education, PIR, marital status), lifestyle behaviors (like smoking, body mass index [BMI], and alcohol status), chronic diseases associated with renal disease (hypertension, cancer, diabetes mellitus, anemia, and hyperuricemia), and biochemical indicators of bone metabolism (calcium and phosphorus). Participants aged 20 years or older were included, categorized by age as young (20-44 years), middle-aged (45-64 years), and elderly (≥65 years). Ethnicity categories included Mexican American, Other Hispanic, Non-Hispanic White, Non-Hispanic Black, and Other Race (including Multi-Racial). Marital status was classified as married/living with a partner, never married, and widowed/divorced. Educational status was categorized as below high school, high school graduate, and college or above. PIR was categorized as <1, 1-1.99, 2-3.99, and ≥4. Smoking status was categorized into three groups: current smokers (those who smoked every day or intermittently), former smokers (those who smoked more than 100 cigarettes in their lifetime but were currently non-smokers), and never smokers (those who smoked less than 100 cigarettes in their lifetime). BMI was categorized as low to normal (<25 kg/m²), overweight (25-30 kg/m²), and obese (≥30 kg/m²). Alcohol consumption was divided into three categories: nondrinkers (those who drank less than 12 drinks in their lifetime), low to moderate drinkers (those who drank 12 drinks in their lifetime but consumed less than 2 drinks per day for men and less than 1 drink per day for women in the past 12 months), and heavy drinkers (those who drank 12 drinks in their lifetime and consumed more than 2 drinks per day for men and more than 1 drink per day for women in the past 12 months). Hypertension and cancer were defined based on a diagnosis by a healthcare professional. Diabetes was diagnosed if any of the following criteria were met: a previous diagnosis of diabetes, fasting blood glucose ≥ 7 mmol/L, or glycated hemoglobin ≥ 6.5%. Anemia was diagnosed based on a hemoglobin concentration of less than 13 g/dl for men and less than 12 g/dl for women (24). Hyperuricemia was diagnosed based on a uric acid level of more than 7 mg/dL in men and more than 6 mg/dL in women (25).

### 2.5 Statistical analysis

The results of NHANE were collected through multiple sectors and there will inevitably be missing data during the survey, our study performed multiple imputations for data with a missing rate of no more than 10 percent (26). Given NHANE’s complex multistage sampling design, appropriate weighting was applied in our analyses. Group differences were assessed using t-tests or ANOVA. Continuous variables were presented as mean ± standard deviation, while categorical variables were reported as frequencies and weighted percentages. We investigated the association between CLR and CKD prevalence risk using weighted logistic regression models. Additionally, the relationship between CLR and markers of kidney injury in CKD was explored using weighted generalized linear regression models. Model 1 was unadjusted, while Model 2 adjusted for age, sex, race, education, PIR, marital status, smoking status, drinking status, and BMI. Model 3 was further adjusted for hypertension, cancer, diabetes, hyperuricemia, anemia, phosphorus, and calcium, based on Model 2. The non-linear relationships between CLR and CKD prevalence risk, eGFR level, and UACR level were examined using weighted restricted cubic splines (RCS). Additionally, the predictive ability of CLR for CKD was assessed using the receiver operating characteristic (ROC) curve and the calculation of the area under the curve (AUC). The optimal cutoff value (OCV) of CLR was determined based on the maximum Youden’s index.

We conducted stratified analyses using logistic and linear regression, stratifying by sex, age, race, education, PIR, marital status, smoking status, drinking status, BMI, hypertension, cancer, diabetes mellitus, hyperuricemia, and anemia classifications. To validate the robustness of the findings, we conducted four sensitivity analyses: First, stroke and cardiac disease were further included after adjusting for all covariates to control for the potential confounding effect of these systemic inflammation-related conditions. Second, unweighted model analyses were performed to assess whether the results were influenced by the weighted data processing. Third, CKD was redefined using stricter criteria (eGFR < 45 ml/min/1.73 m² or ACR ≥ 30 mg/g). Fourth, the use of prescribed medications was incorporated into the analysis, adjusting for covariates such as anti-inflammatory drugs, lipid-lowering drugs, glucose-lowering drugs, and antihypertensive medications, to control for the potential impact of medications on the association between CLR and CKD. All analyses were conducted using R software (version 4.4.0; www.r-project.org), and statistical significance was defined as P < 0.05.

## 3 Results

### 3.1 Characteristics of study participants

We analyzed data from 13,862 respondents in the NHANES database, spanning the years 1999 to 2010. Within this cohort, 2,449 individuals were identified with CKD, presenting a mean age of 59.63 ± 18.25 years, while 11,413 individuals were identified without CKD, with a mean age of 44.10 ± 15.49 years. Age, sex, race, education, PIR, marital status, smoking status, drinking status, BMI, hypertension, cancer, diabetes mellitus, hyperuricemia, anemia, phosphorus, calcium, eGFR, UACR, and CLR were significantly different between the CKD and non-CKD groups (p<0.005). Detailed weighted and unweighted data for participants with and without CKD are provided in **Table 1** and **Supplementary Table S1**, respectively.

**Table 1:** Participant characteristics.

	CKD (N=2449)	non-CKD (N=11413)	P-value
Sex, n (%)			<0.001
female	1289 (56.4%)	5912 (51.0%)	
male	1160 (43.6%)	5501 (49.0%)	
Age, n (%)			<0.001
20–44	393 (23.2%)	5568 (53.3%)	
45–64	641 (30.6%)	3763 (35.0%)	
≥65	1415 (46.2%)	2082 (11.7%)	
Race/ethnicity, n (%)			<0.001
Mexican American	458 (7.2%)	2438 (7.9%)	
Other Hispanic	130 (4.5%)	822 (5.0%)	
Non-Hispanic White	1216 (68.2%)	5635 (71.2%)	
Non-Hispanic Black	558 (14.8%)	2047 (10.3%)	
Other Race - Including Multi-Racial	87 (5.3%)	471 (5.5%)	
Marriage, n (%)			<0.001
Married/living with partner	1336 (57.1%)	7363 (67.6%)	
Never married	225 (10.9%)	1918 (17.2%)	
Widowed/divorced	888 (32.0%)	2132 (15.3%)	
Education, n (%)			<0.001
below high school	974 (28.3%)	3269 (18.0%)	
college or above	889 (44.3%)	5464 (57.2%)	
high school	586 (27.4%)	2680 (24.9%)	
PIR, n (%)			<0.001
<1	532 (16.0%)	2137 (12.8%)	
1–1.99	790 (28.2%)	2945 (19.6%)	
2–3.99	661 (30.4%)	3147 (30.3%)	
≥4	466 (25.4%)	3184 (37.2%)	
Smoke, n (%)			<0.001
Current smoker	407 (18.3%)	2569 (23.6%)	
Former smoker	1219 (51.0%)	6015 (51.8%)	
Never smoker	823 (30.7%)	2829 (24.7%)	
BMI, n (%)			<0.001
Low to normal (<25)	661 (29.1%)	3564 (34.4%)	
Overweight (25–30)	788 (29.6%)	4090 (34.1%)	
Obese (≥30)	1000 (41.3%)	3759 31.6(%)	
Drink, n (%)			<0.001
Heavy drinker	1361 (53.3%)	6520 (55.7%)	
Low to moderate drinker	679 (31.1%)	3472 (34.2%)	
Nondrinker	409 (15.6%)	1421 (10.2%)	
Chronic disease, n (%)			
Hypertension	1487 (56.3%)	3157 (25.1%)	<0.001
Cancer	393 (16.1%)	840 (7.3%)	<0.001
Diabetes	918 (31.1%)	1233 (7.9%)	<0.001
Anemia	409 (36.5%)	739 (18.0%)	<0.001
Hyperuricemia	902 (12.6%)	1953 (4.2%)	<0.001
eGFR, Mean (SD)	77.52 (29.77)	101.60 (17.70)	<0.001
UACR, Mean (SD)	218.50 (777.85)	7.30 (5.36)	<0.001
P, Mean (SD)	3.68 (0.61)	3.61 (0.53)	0.001
Ca, Mean (SD)	9.43 (0.41)	9.41 (0.35)	0.024
CLR, Mean (SD)	0.38 (0.88)	0.21 (0.45)	<0.001

n is unweighted; Both % and M (SD) are weighted. BMI, body mass index; CKD, chronic kidney disease; CLR: C-reactive protein to lymphocyte ratio; eGFR: estimated glomerular filtration rate; PIR, poverty income ratio; UACR: urinary albumin-to-creatinine ratio.

### 3.2 Associations between CLR and CKD

**Figure 2:**
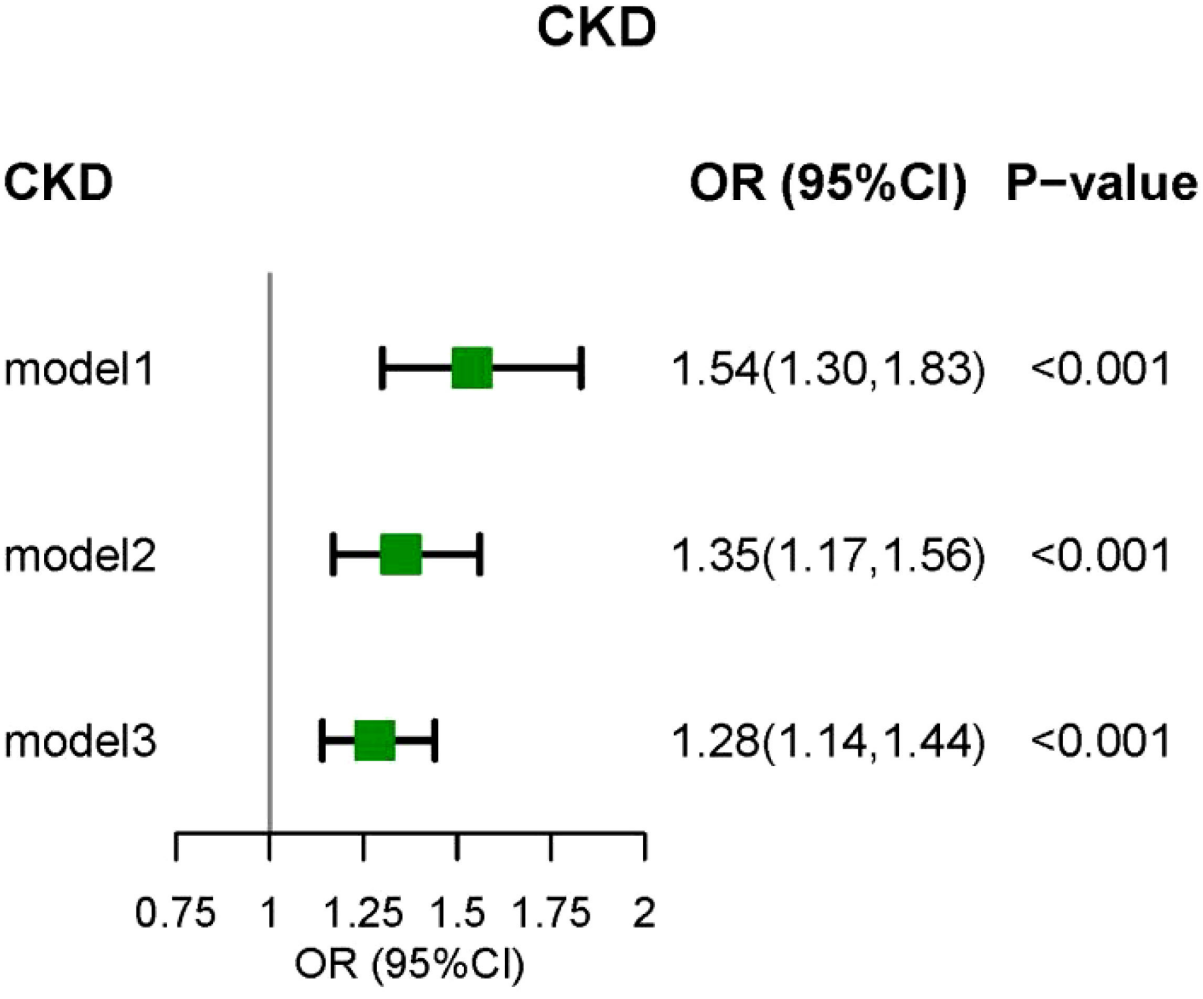
The relationship between CLR and DKD by logistic regression. Model I was the crude model. Multivariable model 2 was adjusted for age, sex, Race/ethnicity, Marital status, Education, PIR, smoking, BMI, and Drink. Multivariable model 3 was adjusted for age, sex, Race/ethnicity, Marital status, Education, PIR, smoking, BMI, Drink, Hypertension, Cancer, Diabetes, Anemia, Hyperuricemia, phosphorus, and calcium. CKD, chronic kidney disease; 95% CI, 95% confidence interval; OR, odds ratio.

**Figure 3:**
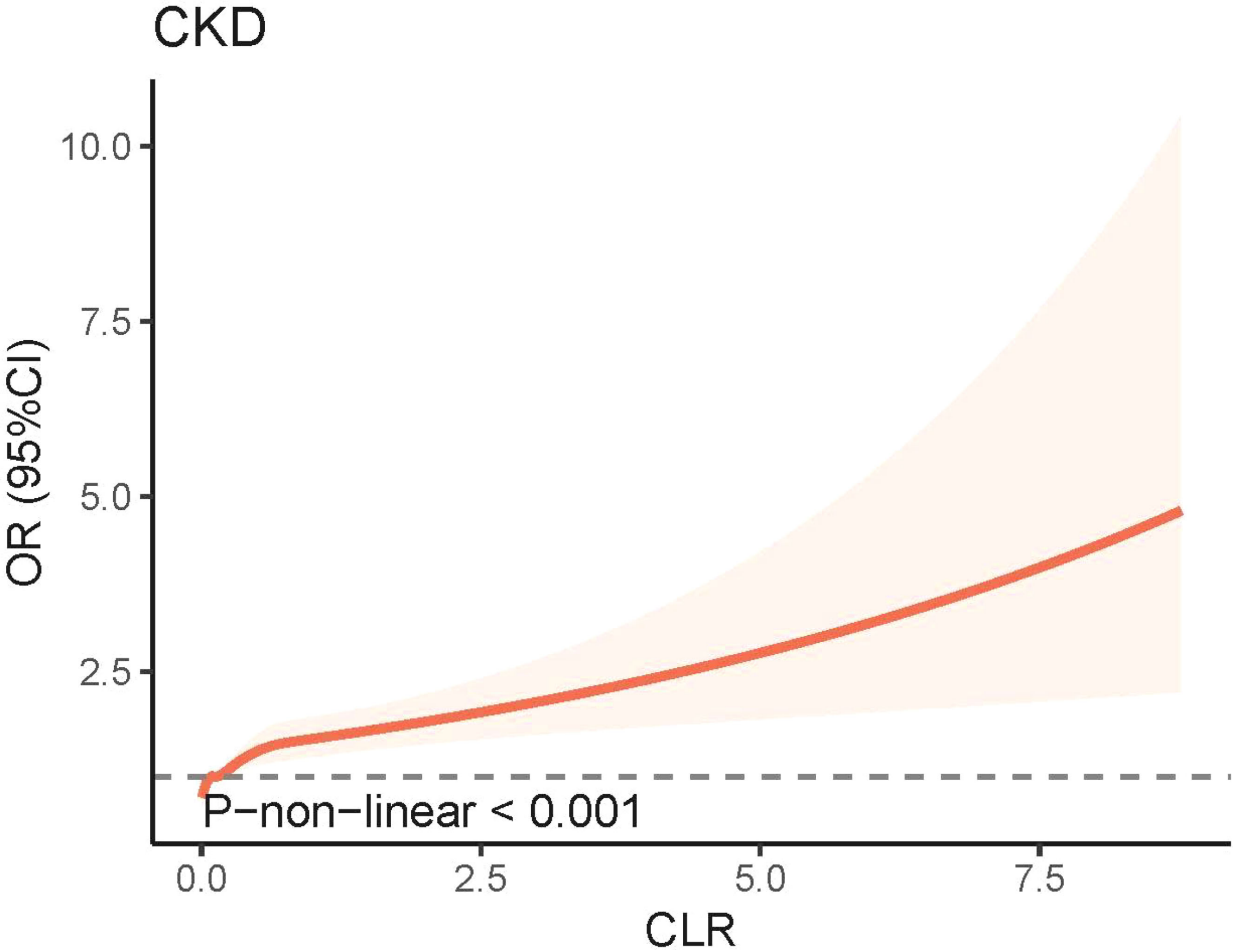
Non-linear association between CLR and CKD by the restricted cubic spline model. CKD, chronic kidney disease; CLR, C-reactive protein to lymphocyte ratio; 95% CI, 95% confidence interval; OR, odds ratio.

The weighted logistic regression analysis revealed a significant association between CKD and CLR. In the unadjusted model, elevated CLR levels were linked to an increased risk of CKD (Odds Ratio [OR] = 1.54, 95% Confidence Interval [CI] = 1.30–1.83, P < 0.001). After adjusting for age, sex, race, education, PIR, marital status, smoking status, drinking status, and BMI in Model 2, CLR remained significantly associated with CKD (OR = 1.35, 95% CI = 1.17–1.56, P < 0.001). In Model 3, which accounted for common CKD complications and various biochemical indicators, the positive correlation between CLR and CKD persisted (**Figure 2**). The RCS model demonstrated a non-linear relationship between CLR and CKD prevalence; however, the graphical representation suggested a more pronounced linear correlation (**Figure 3**).

### 3.3 Associations between CLR and kidney biomarkers

**Figure 4:**
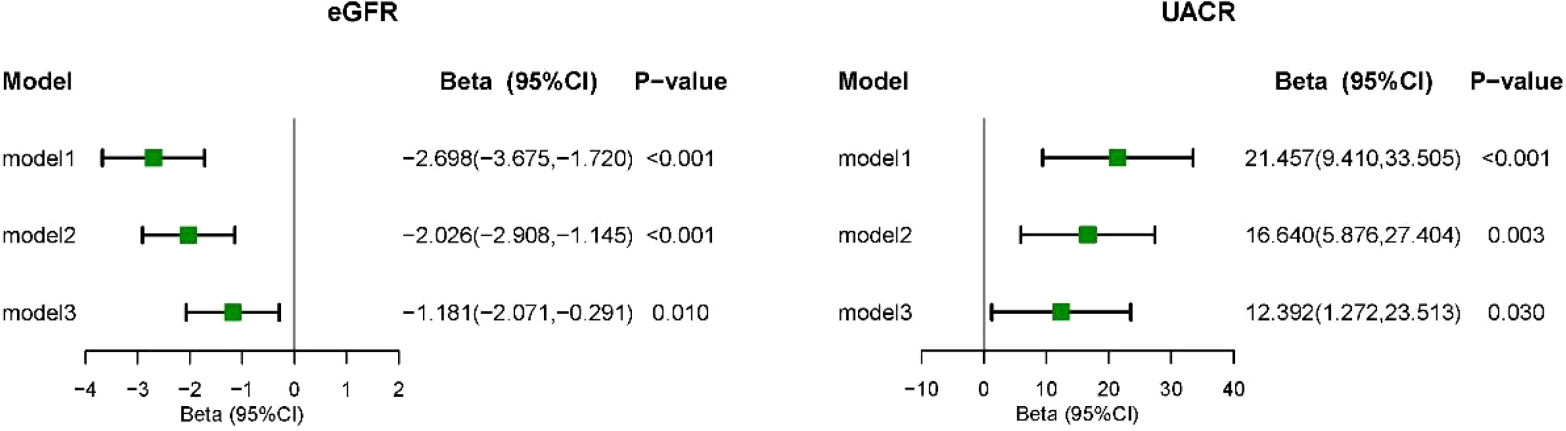
Multivariate linear regression analysis of CLR with kidney biomarkers. Model I was the crude model. Multivariable model 2 was adjusted for age, sex, Race/ethnicity, Marital status, Education, PIR, smoking, BMI, and Drink. Multivariable model 3 was adjusted for age, sex, Race/ethnicity, Marital status, Education, PIR, smoking, BMI, Drink, Hypertension, Cancer, Diabetes, Anemia, Hyperuricemia, P and Ca. 95% CI, 95% confidence interval; eGFR, estimated glomerular filtration rate; UACR, urinary albumin-to-creatinine ratio.

**Figure 5:**
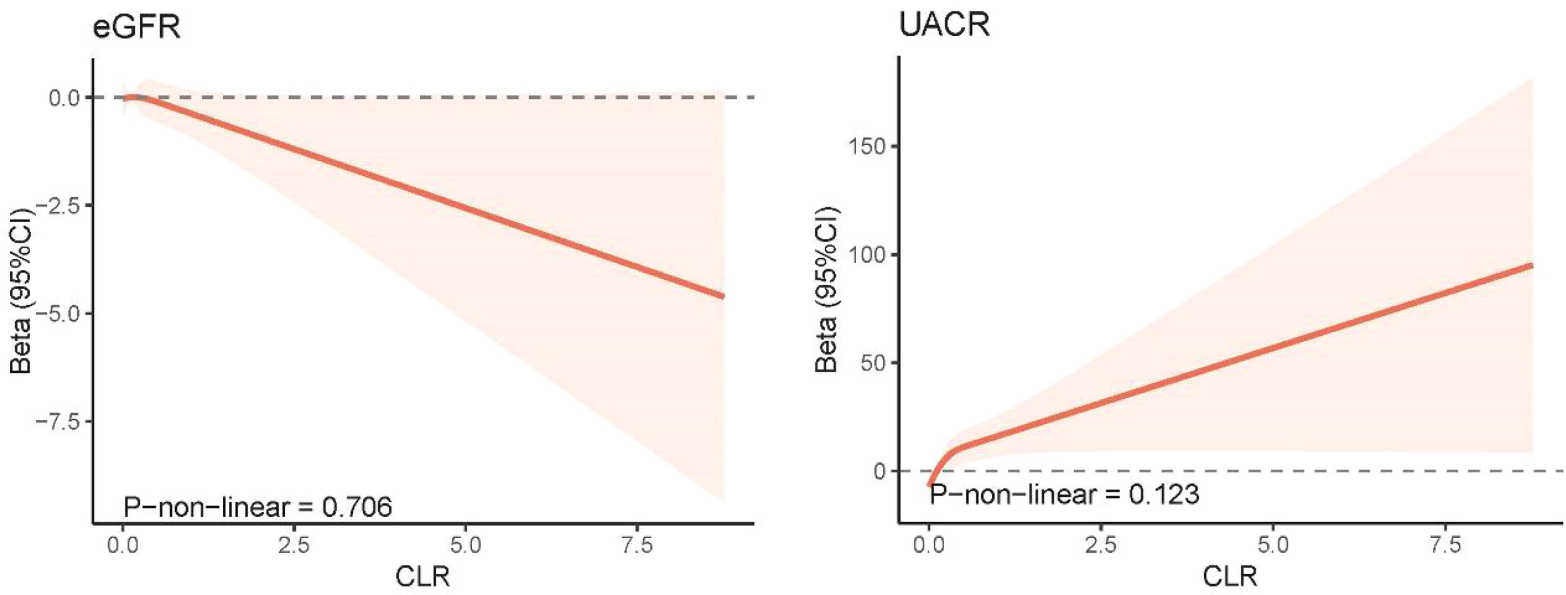
Non-linear association between CLR and kidney biomarkers by the restricted cubic spline model. 95% CI, 95% confidence interval; eGFR, estimated glomerular filtration rate; UACR, urinary albumin-to-creatinine ratio; CLR, C-reactive protein to lymphocyte ratio.

**Figure 4** illustrates the correlation between CLR levels and markers of kidney injury (eGFR and UACR) using survey-weighted multiple linear regression analysis. In Model 3, which adjusted for demographic information, lifestyle habits, chronic conditions associated with kidney disease, and biochemical indicators, CLR was found to be negatively associated with eGFR (β = -1.181, 95% CI = -2.071 to -0.291, P = 0.010). Conversely, the fully adjusted model demonstrated a positive correlation between CLR levels and UACR levels (β = 12.392, 95% CI = 1.272 to 23.513, P = 0.030). The RCS model showed no significant non-linear correlation between CLR and markers of kidney injury (eGFR and UACR) (**Figure 5**).

### 3.4 CLR as a predictor of CKD

**Figure 6:**
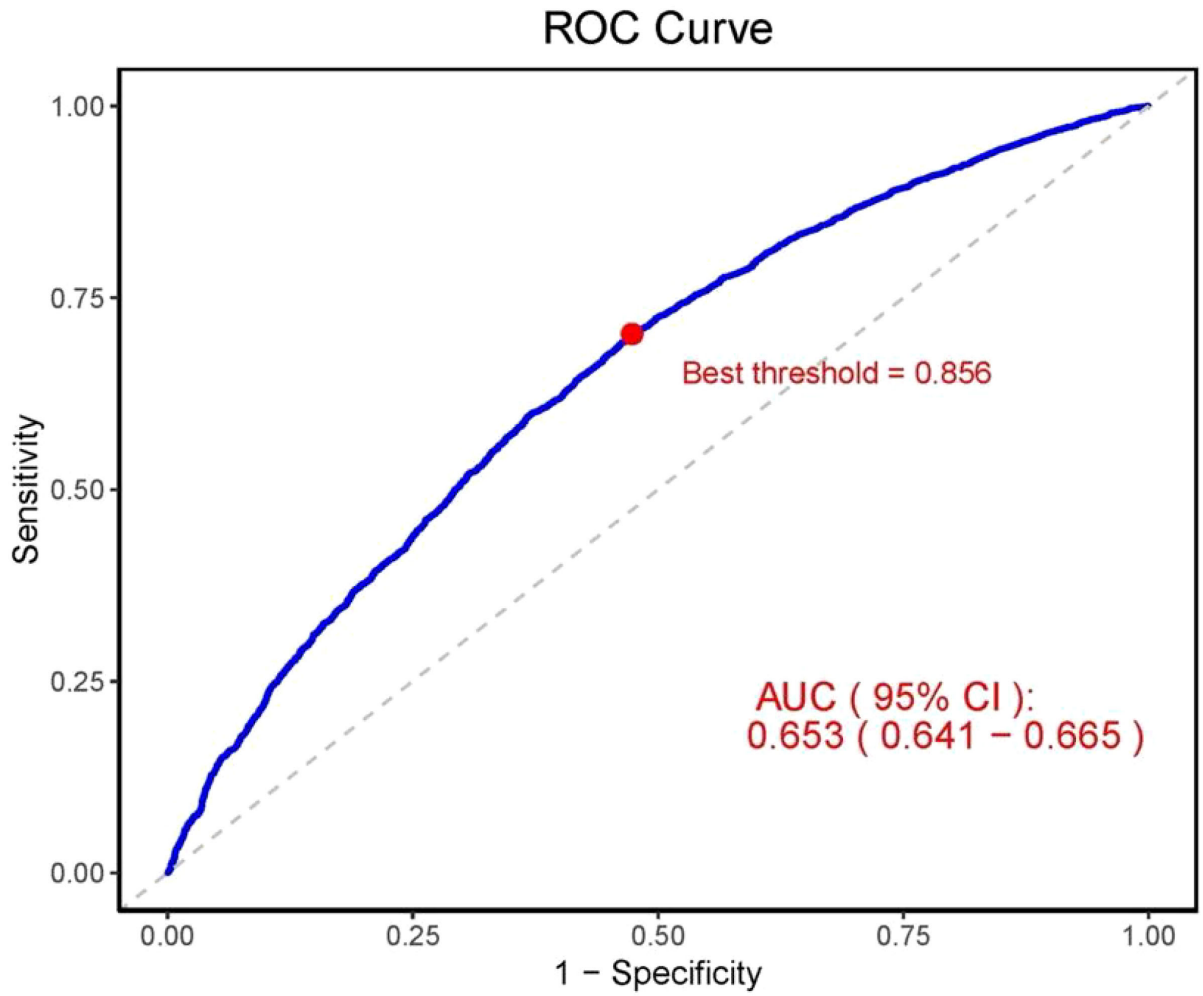
ROC curve for evaluating the predictive ability of CLR for CKD. AUC, area under the curve; 95% CI, 95% confidence interval; ROC, receiver operating characteristic.

To evaluate the predictive ability of CLR for CKD, we utilized the ROC curve and calculated the AUC. The results indicated that the AUC of CLR in predicting CKD was 0.653 (95% CI, 0.641–0.665), suggesting that CLR has a moderate predictive ability in distinguishing between CKD patients and non-CKD individuals (**Figure 6**). The OCV, determined using the maximum Youden’s index method, was 0.856, at which point the sensitivity was 0.703 and the specificity was 0.526. Furthermore, the positive predictive value at the OCV was 0.874, while the negative predictive value was 0.275.

### 3.5 Subgroup analyses

**Figure 7:**
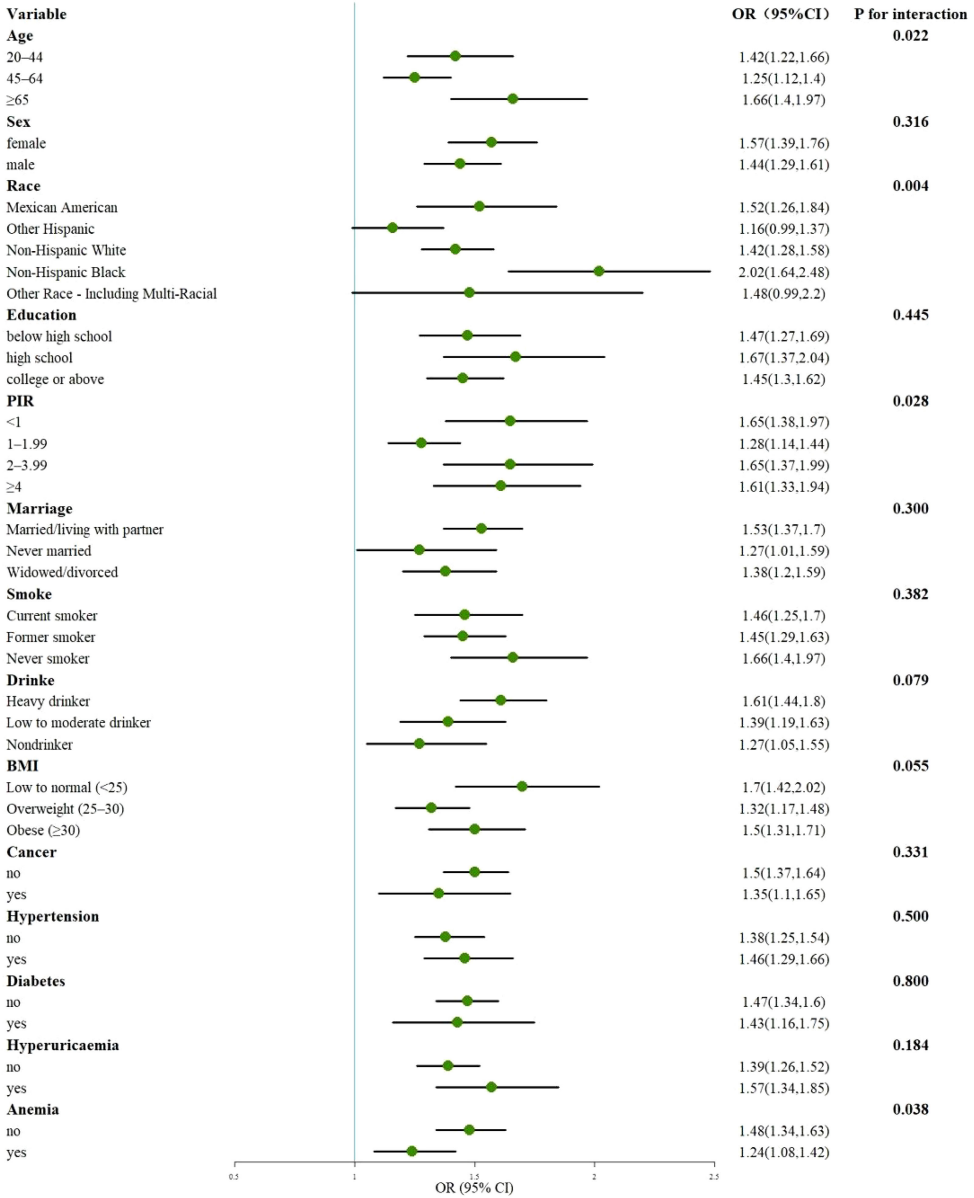
Subgroup analysis of the association of CLR with CKD. OR, odds ratio; 95% CI, 95% confidence interval; PIR, poverty income ratio; BMI, body mass index.

**Figure 7** presents a subgroup analysis of the relationship between CLR and CKD, demonstrating a significant association across all strata, except for other Hispanics and other Race-Including Multi-Racial (P < 0.05). Interaction tests revealed that age, race, PIR, and anemia modified the association between CLR and CKD (P < 0.05). **Supplementary Figure S1** details a subgroup analysis of the relationship between CLR and eGFR. After excluding Age 20-44, other Hispanic, other Race-Including Multi-Racial, Married/living with a partner, PIR ≥ 4, Obese (BMI ≥ 30), Nondrinker, Patients with Cancer, Patients with Diabetes, and Patients without Hypertension, the association between CLR and eGFR remained significant (P < 0.05) across all strata. Interaction tests indicated that gender, age, race, education, PIR, marital status, smoking status, BMI, hypertension, and hyperuricemia influenced the association. **Supplementary Figure S2** performs subgroup analyses of the relationship between CLR and UACR. After removing Age ≥ 65, Mexican American, other Hispanic, other Race-Including Multi-Racial, Never married, college or above, PIR 2-3.99, Current smoker, Low to moderate drinker, and Nondrinker, the relationship between CLR and UACR was significant (P < 0.05) in all strata. Interaction tests showed that race and cancer affected the association.

### 3.6 Sensitivity analysis

**Supplementary Tables S8**-**S19** present the results of the sensitivity analysis, where we employed four methods to assess the robustness of the findings. First, after adjusting for all covariates (including age, sex, BMI, hypertension, diabetes, etc.), and further including stroke and heart disease variables, the results indicated that the associations between CLR and CKD, eGFR, and UACR remained significant (OR = 0.78, 95% CI, 0.70–0.88, P < 0.001; β = -0.978, 95% CI, -1.848 to -0.108, P = 0.028; β = 11.768, 95% CI, 0.830–22.706, P = 0.035). Second, unweighted model analysis was performed to assess whether the results were influenced by the weighted data processing, and it showed that the association between CLR and CKD, as well as CLR and eGFR, remained significant (OR = 0.81, 95% CI, 0.75–0.87, P < 0.001; β = -1.144, 95% CI, -1.666 to -0.623, P < 0.001). However, the association between CLR and UACR approached the significance level (β = 11.744, 95% CI, -0.019 to 23.506, P = 0.050). Third, CKD was redefined using stricter diagnostic criteria (eGFR < 45 ml/min/1.73 m² or ACR ≥ 30 mg/g), and the results of these analyses were largely consistent with the original findings. Finally, after adjusting for all covariates and incorporating the use of prescribed medications (including anti-inflammatory drugs, lipid-lowering drugs, glucose-lowering drugs, and antihypertensive medications), the association between CLR and CKD, as well as CLR and eGFR, remained significant (OR = 0.78, 95% CI, 0.69–0.88, P < 0.001; β = -1.101, 95% CI, -1.943 to -0.259, P = 0.011). However, the association between CLR and UACR was no longer significant (P > 0.05). These sensitivity analyses suggest that CLR has strong robustness in its correlation with CKD and eGFR, but its predictive ability for UACR may be influenced by factors such as pharmacological interventions.

## 4 Discussion

Our study analyzed the NHANES database and identified a significant nonlinear and positive association between CLR and the prevalence of CKD in a representative population of US adults. There was a significant negative correlation between CLR and eGFR, and a significant positive correlation with UACR. ROC curves show the predictive power of CLR in differentiating CKD patients from non-CKD patients. Sensitivity analyses and subgroup analyses demonstrated the robustness and reliability of the results. These findings suggest that CLR may serve as an inflammatory marker in the onset and progression of CKD. Therefore, controlling CLR levels might play a role in improving the clinical management of CKD.

Inflammation constitutes a fundamental component of the body’s defense mechanisms, categorized into acute and chronic forms. Chronic inflammation plays a pivotal role in the morbidity and mortality associated with CKD, serving as a key pathogenic mechanism (27). This process primarily involves the infiltration of inflammatory cells and the production of inflammatory cytokines, which promote tissue damage (28). Chronic inflammation in CKD arises from various causes (29, 30). On one hand, the decline in the eGFR in CKD diminishes the kidney’s capacity to effectively filter toxins and harmful substances, resulting in their accumulation. These endotoxins stimulate the production of inflammatory mediators such as interleukins and tumor necrosis factor, thereby exacerbating inflammatory states (31, 32). On the other hand, oxidative stress, activated during CKD progression, synergistically interacts with inflammation, further aggravating kidney damage (33). Additionally, alterations in intestinal flora due to enterotoxins (34, 35), compromised immune function affecting monocytes and lymphocytes (36), and infections associated with dialysis (37)collectively contribute to the development of chronic inflammation in CKD. Consequently, recent research has focused on monitoring and investigating inflammatory markers in kidney disease.

CRP is a pivotal acute-phase protein and a classical marker of systemic inflammation. It is predominantly produced by inflammatory cells and can also be expressed by renal tubular epithelial and endothelial cells. Elevated serum CRP levels have been associated with an increased risk of CKD (38). Animal studies have demonstrated that mice with high expression of the human CRP gene exhibit heightened susceptibility to renal inflammation and fibrosis, potentially through the activation of the NF-κB and TGF-β/Smad signaling pathways (39). A reduction in B and T lymphocytes has been implicated in the development of atherosclerosis in CKD patients (40). Xiong et al. found that CKD patients exhibit defective T-lymphocyte subpopulations, which correlate with infections and renal outcomes in these patients (41). CLR is the ratio of CRP to lymphocytes and responds to the inflammatory and immune state of the body. CLR has emerged as a significant marker in diagnosing and prognosticating infections, tumors, neo-coronary conditions, dilated cardiomyopathy, and other diseases. A low CLR level has been linked to longer progression-free survival in breast cancer (42), whereas elevated CLR is associated with poor prognosis in pancreatic cancer patients (43). CLR could also serve as a marker for hepatitis C infection and its elevation may be indicative of liver fibrosis (44). Furthermore, CLR has utility as a screening tool for diagnosing periprosthetic arthritis infections and postoperative infections such as those following lumbar spine surgeries (45). Additionally, CLR can be utilized as a biomarker for early screening and predicting the severity of novel coronavirus infections and associated mortality (46). However, the role of CLR in CKD is less well-studied. Our study explored the potential of CLR as a novel biomarker for predicting the prevalence of CKD, reflecting both chronic inflammation and immune dysregulation in CKD patients, compared to traditional markers such as CRP, cytokines, TNF receptor-1, TNF receptor-2, and other individual markers (47, 48). Additionally, since CRP and peripheral blood lymphocyte counts are commonly used clinical assays, CLR testing is easily accessible and cost-effective, making it a practical option for clinical application.

Our study included 13,862 respondents from the 1999-2010 NHANES database and represents a large, cross-sectional dataset. The study population is based on a nationally representative sample of U.S. adults. Weighted logistic regression models demonstrated a positive association between CLR and CKD, with higher CLR levels predicting a greater prevalence of CKD. Since eGFR is the best available indicator of overall renal function and UACR is closely associated with CKD progression and poor prognosis (49, 50), we further explored the relationship between CLR, eGFR, and UACR. We found that high CLR levels were associated with lower eGFR and higher UACR levels. CKD is frequently comorbid with diabetes, hypertension, and hyperlipidemia, all of which are also risk factors for CKD (51, 52). After adjusting for these confounders, we conducted additional sensitivity analyses accounting for medications such as anti-inflammatory drugs, lipid-lowering agents, glucose-lowering drugs, and antihypertensive medications, all of which may influence serum inflammation levels (53–55). These analyses confirmed that the overall association between CLR and CKD remained significant, further validating the independent role of CLR. However, the association between CLR and UACR changed from statistically significant (P < 0.05) to non-significant (P > 0.05) after adjusting for prescribed medications. This suggests that UACR may be more susceptible to pharmacological interventions, particularly lipid-lowering and glucose-lowering drugs, which might reduce CLR’s predictive power for UACR by improving systemic metabolism and inflammation (56, 57). This finding highlights a potential differential role of CLR in relation to different CKD indicators, such as eGFR and UACR, and underscores the importance of considering pharmacological interventions when interpreting the relationship between inflammatory markers and CKD. Finally, we used a ROC curve to evaluate CLR’s predictive ability for CKD. The results showed that CLR had a moderate capacity to identify individuals at higher risk for CKD, with a particularly strong positive predictive value but a low negative predictive value, indicating limited ability to identify individuals without CKD. As a potential inflammatory marker, CLR could provide valuable insight for early CKD screening, though its practical application would be most effective when combined with other clinical indicators. However, there are several limitations to this study. Firstly, the study population was primarily from the United States, which may limit the generalizability of our findings to other populations. Secondly, being a cross-sectional study, we cannot establish causality. Future large-scale clinical studies and animal experiments are needed to further validate our findings.

## 5 Conclusions

In this large-scale cross-sectional study of the US adult population, we observed a positive association between CLR and the prevalence of CKD. Concurrently, CLR exhibited a negative correlation with eGFR and a positive correlation with UACR. These findings suggest that CLR holds promise as a potential biomarker for guiding the development, prognosis, and treatment of CKD. However, further prospective clinical and animal studies are essential to validate these associations.

## Data Availability

The raw data supporting the conclusions of this article will be made available by the authors, without undue reservation.
