# Supplementary material for: The relationship between C-reactive protein to lymphocyte ratio and the prevalence of chronic kidney disease in US adults: a cross-sectional study
Source: Front Endocrinol (Lausanne). 2025 Jan 15;15:1469750. doi: 10.3389/fendo.2024.1469750 (PMC11774709; doi:10.3389/fendo.2024.1469750)
Supplement: Supplementary file 1 [file DataSheet1.docx]

Supplementary Material

# Supplementary Tables

**Supplementary table S1. unweighted baseline table**

|  | **CKD (N=2449)** | **non-CKD (N=11413)** | **P-value** |
| --- | --- | --- | --- |
| **Sex, n (%)** |  |  | 0.756 |
| female | 1289 (52.6%) | 5912 (51.8%) |  |
| male | 1160 (47.4%) | 5501 (48.2%) |  |
| **Age, n (%)** |  |  | <0.001 |
| 20–44 | 393 (16.0%) | 5568 (48.8%) |  |
| 45–64 | 641 (26.2%) | 3763 (33.0%) |  |
| ≥65 | 1415 (57.8%) | 2082 (18.2%) |  |
| **Race/ethnicity, n (%)** |  |  | <0.001 |
| Mexican American | 458 (18.7%) | 2438 (21.4%) |  |
| Other Hispanic | 130 (5.3%) | 822 (7.2%) |  |
| Non-Hispanic White | 1216 (49.7%) | 5635 (49.4%) |  |
| Non-Hispanic Black | 558 (22.8%) | 2047 (17.9%) |  |
| Other Race - Including Multi-Racial | 87 (3.6%) | 471 (4.1%) |  |
| **Marriage, n (%)** |  |  | <0.001 |
| Married/living with partner | 1336 (54.6%) | 7363 (64.5%) |  |
| Never married | 225 (9.2%) | 1918 (16.8%) |  |
| Widowed/divorced | 888 (36.3%) | 2132 (18.7%) |  |
| **Education, n (%)** |  |  | <0.001 |
| below high school | 974 (39.8%) | 3269 (28.6%) |  |
| college or above | 889 (36.3%) | 5464 (47.9%) |  |
| high school | 586 (23.9%) | 2680 (23.5%) |  |
| **PIR, n (%)** |  |  | <0.001 |
| <1 | 532 (21.7%) | 2137 (18.7%) |  |
| 1–1.99 | 790 (32.3%) | 2945 (25.8%) |  |
| 2–3.99 | 661 (27.0%) | 3147 (27.6%) |  |
| ≥4 | 466 (19.0%) | 3184 (27.9%) |  |
| **Smoke, n (%)** |  |  | <0.001 |
| Current smoker | 407 (16.6%) | 2569 (22.5%) |  |
| Former smoker | 1219 (49.8%) | 6015 (52.7%) |  |
| Never smoker | 823 (33.6%) | 2829 (24.8%) |  |
| **BMI, n (%)** |  |  | <0.001 |
| Low to normal (<25) | 661 (27.0%) | 3564 (31.2%) |  |
| Overweight (25–30) | 788 (32.2%) | 4090 (35.8%) |  |
| Obese (≥30) | 1000 (40.8%) | 3759 (32.9%) |  |
| **Drink, n (%)** |  |  | <0.001 |
| Heavy drinker | 1361 (55.6%) | 6520 (57.1%) |  |
| Low to moderate drinker | 679 (27.7%) | 3472 (30.4%) |  |
| Nondrinker | 409 (16.7%) | 1421 (12.5%) |  |
| **Chronic disease, n (%)** |  |  |  |
| **Hypertension** | 1487 (60.7%) | 3157 (27.7%) | <0.001 |
| **Cancer** | 393 (16.0%) | 840 (7.4%) | <0.001 |
| **Diabetes** | 918 (37.5%) | 1233 (10.8%) | <0.001 |
| **Anemia** | 409 (16.7%) | 739 (6.5%) | <0.001 |
| **Hyperuricaemia** | 902 (36.8%) | 1953 (17.1%) | <0.001 |
| **eGFR, Mean (SD)** | 73.1 (29.3) | 101 (18.8) | <0.001 |
| **UACR, Mean (SD)** | 273 (1010) | 7.90 (5.70) | <0.001 |
| **P, Mean (SD)** | 3.65 (0.601) | 3.62 (0.532) | 0.135 |
| **Ca, Mean (SD)** | 9.42 (0.421) | 9.39 (0.364) | 0.00111 |
| **CLR, Mean (SD)** | 0.405 (0.983) | 0.235 (0.504) | <0.001 |

BMI, body mass index; CKD, chronic kidney disease; CLR, C-reactive protein to lymphocyte ratio; eGFR: estimated glomerular filtration rate; PIR, poverty income ratio; UACR, urinary albumin-to-creatinine ratio.

**Supplementary Table S2 CLR and CKD incidence modle2**

| **Characteristic** | **OR***^1^* | **95% CI***^1^* | **p-value** |
| --- | --- | --- | --- |
| **Age** |  |  |  |
| ≥65 | — | — |  |
| 20–44 | 0.11 | 0.09, 0.13 | <0.001 |
| 45–64 | 0.24 | 0.20, 0.27 | <0.001 |
| **Sex** |  |  |  |
| female | — | — |  |
| male | 1.02 | 0.90, 1.16 | 0.8 |
| **Race** |  |  |  |
| Mexican American | — | — |  |
| Other Hispanic | 0.80 | 0.60, 1.09 | 0.2 |
| Non-Hispanic White | 0.83 | 0.70, 0.98 | 0.030 |
| Non-Hispanic Black | 1.34 | 1.12, 1.59 | 0.001 |
| Other Race - Including Multi-Racial | 1.16 | 0.84, 1.61 | 0.4 |
| **Education** |  |  |  |
| below high school | — | — |  |
| college or above | 0.78 | 0.65, 0.94 | 0.011 |
| high school | 0.90 | 0.76, 1.07 | 0.2 |
| **PIR** |  |  |  |
| <1 | — | — |  |
| 1–1.99 | 0.96 | 0.79, 1.17 | 0.7 |
| 2–3.99 | 0.82 | 0.66, 1.03 | 0.082 |
| ≥4 | 0.69 | 0.55, 0.87 | 0.002 |
| **Marriage** |  |  |  |
| Married/living with partner | — | — |  |
| Never married | 1.18 | 0.93, 1.50 | 0.2 |
| Widowed/divorced | 1.47 | 1.26, 1.72 | <0.001 |
| **Smoke** |  |  |  |
| Current smoker | — | — |  |
| Former smoker | 1.11 | 0.93, 1.32 | 0.3 |
| Never smoker | 1.00 | 0.83, 1.21 | >0.9 |
| **Drink** |  |  |  |
| Heavy drinker | — | — |  |
| Low to moderate drinker | 0.87 | 0.73, 1.03 | 0.11 |
| Nondrinker | 1.03 | 0.83, 1.29 | 0.8 |
| **BMI** |  |  |  |
| Low to normal (<25) | — | — |  |
| Obese (≥30) | 1.37 | 1.16, 1.61 | <0.001 |
| Overweight (25–30) | 0.88 | 0.75, 1.04 | 0.14 |
| **CLR** | 1.35 | 1.17, 1.56 | <0.001 |

Multivariable model 2 was adjusted for age, sex, Race/ethnicity, Marital status, Education, PIR, Smoke, BMI and Drinke.

BMI, body mass index; CKD, chronic kidney disease; CLR, C-reactive protein to lymphocyte ratio; OR, odds ratio; PIR, poverty income ratio; 95% CI, 95% confidence interval.

**Supplementary Table S3 CLR and CKD incidence modle3**

| **Characteristic** | **OR** | **95% CI** | **p-value** |
| --- | --- | --- | --- |
| **Age** |  |  |  |
| ≥65 | — | — |  |
| 20–44 | 0.20 | 0.16, 0.24 | <0.001 |
| 45–64 | 0.30 | 0.26, 0.36 | <0.001 |
| **Sex** |  |  |  |
| female | — | — |  |
| male | 1.00 | 0.87, 1.14 | >0.9 |
| **Race** |  |  |  |
| Mexican American | — | — |  |
| Other Hispanic | 0.78 | 0.58, 1.06 | 0.11 |
| Non-Hispanic White | 0.78 | 0.66, 0.92 | 0.003 |
| Non-Hispanic Black | 1.03 | 0.85, 1.24 | 0.8 |
| Other Race - Including Multi-Racial | 0.94 | 0.67, 1.31 | 0.7 |
| **Education** |  |  |  |
| below high school | — | — |  |
| college or above | 0.82 | 0.67, 1.01 | 0.056 |
| high school | 0.91 | 0.76, 1.09 | 0.3 |
| **PIR** |  |  |  |
| <1 | — | — |  |
| 1–1.99 | 0.97 | 0.79, 1.19 | 0.8 |
| 2–3.99 | 0.85 | 0.67, 1.07 | 0.2 |
| ≥4 | 0.73 | 0.58, 0.92 | 0.009 |
| **Marriage** |  |  |  |
| Married/living with partner | — | — |  |
| Never married | 1.18 | 0.92, 1.51 | 0.2 |
| Widowed/divorced | 1.40 | 1.19, 1.66 | <0.001 |
| **Smoke** |  |  |  |
| Current smoker | — | — |  |
| Former smoker | 1.08 | 0.90, 1.29 | 0.4 |
| Never smoker | 0.93 | 0.76, 1.13 | 0.4 |
| **Drink** |  |  |  |
| Heavy drinker | — | — |  |
| Low to moderate drinker | 0.92 | 0.77, 1.10 | 0.3 |
| Nondrinker | 0.97 | 0.77, 1.23 | 0.8 |
| **BMI** |  |  |  |
| Low to normal (<25) | — | — |  |
| Obese (≥30) | 0.83 | 0.68, 1.02 | 0.076 |
| Overweight (25–30) | 0.75 | 0.63, 0.91 | 0.003 |
| **Cancer** |  |  |  |
| no | — | — |  |
| yes | 1.15 | 0.91, 1.46 | 0.2 |
| **Hypertension** |  |  |  |
| no | — | — |  |
| yes | 1.81 | 1.56, 2.11 | <0.001 |
| **Diabetes** |  |  |  |
| yes | — | — |  |
| no | 0.38 | 0.31, 0.46 | <0.001 |
| **Hyperuricaemia** |  |  |  |
| no | — | — |  |
| yes | 1.86 | 1.57, 2.21 | <0.001 |
| **Anemia** |  |  |  |
| no | — | — |  |
| yes | 1.99 | 1.59, 2.50 | <0.001 |
| **P** | 1.13 | 0.97, 1.32 | 0.11 |
| **Ca** | 1.02 | 0.83, 1.25 | 0.9 |
| **CLR** | 1.28 | 1.14, 1.44 | <0.001 |

Multivariable model 3 was adjusted for age, sex, Race/ethnicity, Marital status, Education, PIR, Smoke, BMI, Drink, Hypertension, Cancer, Diabetes, Anemia, Hyperuricaemia, P and Ca.

BMI, body mass index; CKD, chronic kidney disease; CLR, C-reactive protein to lymphocyte ratio; OR, odds ratio; PIR, poverty income ratio; 95% CI, 95% confidence interval.

**Supplementary Table S4 CLR and eGFR levels of modle2**

| **Characteristic** | **Beta** | **95% CI** | **p-value** |
| --- | --- | --- | --- |
| **Age** |  |  |  |
| ≥65 | — | — |  |
| 20–44 | 34.457 | 33.352, 35.563 | <0.001 |
| 45–64 | 19.200 | 18.007, 20.392 | <0.001 |
| **Sex** |  |  |  |
| female | — | — |  |
| male | -1.303 | -2.093, -0.513 | 0.002 |
| **Race** |  |  |  |
| Mexican American | — | — |  |
| Other Hispanic | -4.572 | -7.251, -1.892 | 0.001 |
| Non-Hispanic White | -15.079 | -16.857, -13.301 | <0.001 |
| Non-Hispanic Black | -18.121 | -20.266, -15.976 | <0.001 |
| Other Race - Including Multi-Racial | -8.305 | -10.993, -5.617 | <0.001 |
| **Education** |  |  |  |
| below high school | — | — |  |
| college or above | 3.223 | 1.780, 4.666 | <0.001 |
| high school | 1.627 | 0.179, 3.075 | 0.028 |
| **PIR** |  |  |  |
| <1 | — | — |  |
| 1–1.99 | -3.516 | -5.114, -1.917 | <0.001 |
| 2–3.99 | -2.971 | -4.671, -1.271 | <0.001 |
| ≥4 | -3.826 | -5.500, -2.152 | <0.001 |
| **Marriage** |  |  |  |
| Married/living with partner | — | — |  |
| Never married | 8.265 | 7.142, 9.389 | <0.001 |
| Widowed/divorced | -10.216 | -11.487, -8.946 | <0.001 |
| **Smoke** |  |  |  |
| Current smoker | — | — |  |
| Former smoker | -4.368 | -5.615, -3.120 | <0.001 |
| Never smoker | -8.264 | -9.577, -6.952 | <0.001 |
| **Drink** |  |  |  |
| Heavy drinker | — | — |  |
| Low to moderate drinker | -3.854 | -4.801, -2.906 | <0.001 |
| Nondrinker | -4.852 | -6.702, -3.002 | <0.001 |
| **BMI** |  |  |  |
| Low to normal (<25) | — | — |  |
| Obese (≥30) | -2.144 | -3.124, -1.165 | <0.001 |
| Overweight (25–30) | -3.318 | -4.420, -2.216 | <0.001 |
| **CLR** | -2.026 | -2.908, -1.145 | <0.001 |

Multivariable model 2 was adjusted for age, sex, Race/ethnicity, Marital status, Education, PIR, Smoke, BMI, and Drink.

BMI, body mass index; CLR, C-reactive protein to lymphocyte ratio; eGFR, estimated glomerular filtration rate; PIR, poverty income ratio; 95% CI, 95% confidence interval.

**Supplementary Table S5 CLR and eGFR levels of modle3**

| **Characteristic** | **Beta** | **95% CI** | **p-value** |
| --- | --- | --- | --- |
| **Age** |  |  |  |
| ≥65 | — | — |  |
| 20–44 | 31.460 | 30.308, 32.612 | <0.001 |
| 45–64 | 17.201 | 15.997, 18.405 | <0.001 |
| **Sex** |  |  |  |
| female | — | — |  |
| male | -1.256 | -2.065, -0.447 | 0.003 |
| **Race** |  |  |  |
| Mexican American | — | — |  |
| Other Hispanic | -3.405 | -5.835, -0.974 | 0.007 |
| Non-Hispanic White | -12.025 | -13.563, -10.488 | <0.001 |
| Non-Hispanic Black | -14.372 | -16.247, -12.497 | <0.001 |
| Other Race - Including Multi-Racial | -5.420 | -7.730, -3.111 | <0.001 |
| **Education** |  |  |  |
| below high school | — | — |  |
| college or above | 1.927 | 0.665, 3.188 | 0.003 |
| high school | 1.240 | -0.002, 2.482 | 0.050 |
| **PIR** |  |  |  |
| <1 | — | — |  |
| 1–1.99 | -2.923 | -4.252, -1.595 | <0.001 |
| 2–3.99 | -2.879 | -4.412, -1.345 | <0.001 |
| ≥4 | -3.662 | -5.108, -2.217 | <0.001 |
| **Marriage** |  |  |  |
| Married/living with partner | — | — |  |
| Never married | 7.270 | 6.173, 8.367 | <0.001 |
| Widowed/divorced | -7.893 | -8.999, -6.786 | <0.001 |
| **Smoke** |  |  |  |
| Current smoker | — | — |  |
| Former smoker | -4.033 | -5.238, -2.827 | <0.001 |
| Never smoker | -6.355 | -7.637, -5.073 | <0.001 |
| **Drink** |  |  |  |
| Heavy drinker | — | — |  |
| Low to moderate drinker | -3.502 | -4.427, -2.576 | <0.001 |
| Nondrinker | -3.378 | -5.028, -1.728 | <0.001 |
| **BMI** |  |  |  |
| Low to normal (<25) | — | — |  |
| Obese (≥30) | 1.775 | 0.860, 2.690 | <0.001 |
| Overweight (25–30) | -2.059 | -3.145, -0.974 | <0.001 |
| **Cancer** |  |  |  |
| no | — | — |  |
| yes | -9.268 | -10.764, -7.772 | <0.001 |
| **Hypertension** |  |  |  |
| no | — | — |  |
| yes | -9.310 | -10.271, -8.349 | <0.001 |
| **Diabetes** |  |  |  |
| yes | — | — |  |
| no | 4.361 | 3.014, 5.707 | <0.001 |
| **Hyperuricaemia** |  |  |  |
| no | — | — |  |
| yes | -7.355 | -8.657, -6.052 | <0.001 |
| **Anemia** |  |  |  |
| no | — | — |  |
| yes | -5.235 | -7.069, -3.400 | <0.001 |
| **P** | -3.104 | -4.044, -2.164 | <0.001 |
| **Ca** | -3.648 | -5.232, -2.064 | <0.001 |
| **CLR** | -1.181 | -2.071, -0.291 | 0.010 |

Multivariable model 3 was adjusted for age, sex, Race/ethnicity, Marital status, Education, PIR, Smoke, BMI, Drink, Hypertension, Cancer, Diabetes, Anemia, Hyperuricaemia, P and Ca.

BMI, body mass index; CLR, C-reactive protein to lymphocyte ratio; eGFR: estimated glomerular filtration rate; PIR , poverty income ratio; 95% CI, 95% confidence interval.

**Supplementary Table S6 CLR and UACR levels of modle2**

| **Characteristic** | **Beta** | **95% CI** | **p-value** |
| --- | --- | --- | --- |
| **Age** |  |  |  |
| ≥65 | — | — |  |
| 20–44 | -41.633 | -52.671, -30.596 | <0.001 |
| 45–64 | -10.739 | -25.571, 4.092 | 0.153 |
| **Sex** |  |  |  |
| female | — | — |  |
| male | 7.970 | -2.799, 18.740 | 0.144 |
| **Race** |  |  |  |
| Mexican American | — | — |  |
| Other Hispanic | 1.052 | -28.408, 30.512 | 0.943 |
| Non-Hispanic White | -10.644 | -24.406, 3.118 | 0.127 |
| Non-Hispanic Black | 18.081 | -7.932, 44.094 | 0.170 |
| Other Race - Including Multi-Racial | 15.051 | -12.399, 42.501 | 0.278 |
| **Education** |  |  |  |
| below high school | — | — |  |
| college or above | -2.536 | -18.900, 13.827 | 0.758 |
| high school | 2.175 | -14.690, 19.040 | 0.798 |
| **PIR** |  |  |  |
| <1 | — | — |  |
| 1–1.99 | -15.591 | -41.099, 9.917 | 0.227 |
| 2–3.99 | -26.282 | -49.510, -3.054 | 0.027 |
| ≥4 | -34.808 | -59.614, -10.003 | 0.007 |
| **Marriage** |  |  |  |
| Married/living with partner | — | — |  |
| Never married | 0.157 | -9.663, 9.977 | 0.975 |
| Widowed/divorced | 14.738 | -1.597, 31.072 | 0.076 |
| **Smoke** |  |  |  |
| Current smoker | — | — |  |
| Former smoker | 5.253 | -7.363, 17.869 | 0.409 |
| Never smoker | 8.385 | -7.252, 24.022 | 0.289 |
| **Drinke** |  |  |  |
| Heavy drinker | — | — |  |
| Low to moderate drinker | -11.464 | -22.169, -0.760 | 0.036 |
| Nondrinker | -7.743 | -25.696, 10.211 | 0.393 |
| **BMI** |  |  |  |
| Low to normal (<25) | — | — |  |
| Obese (≥30) | 8.732 | -2.211, 19.674 | 0.116 |
| Overweight (25–30) | -4.430 | -14.726, 5.866 | 0.393 |
| **CLR** | 16.640 | 5.876, 27.404 | 0.003 |

Multivariable model 2 was adjusted for age, sex, Race/ethnicity, Marital status, Education, PIR, Smoke, BMI and Drink.

BMI, body mass index; CLR, C-reactive protein to lymphocyte ratio; PIR, poverty income ratio; UACR, urinary albumin-to-creatinine ratio; 95% CI, 95% confidence interval.

**Supplementary Table S7 CLR and UACR levels of modle3**

| **Characteristic** | **Beta** | **95% CI** | **p-value** |
| --- | --- | --- | --- |
| **Age** |  |  |  |
| ≥65 | — | — |  |
| 20–44 | -3.735 | -21.109, 13.638 | 0.669 |
| 45–64 | 11.013 | -9.918, 31.944 | 0.297 |
| **Sex** |  |  |  |
| female | — | — |  |
| male | 10.430 | -2.900, 23.759 | 0.123 |
| **Race** |  |  |  |
| Mexican American | — | — |  |
| Other Hispanic | -0.086 | -29.252, 29.080 | 0.995 |
| Non-Hispanic White | -10.058 | -24.561, 4.446 | 0.171 |
| Non-Hispanic Black | 7.866 | -17.465, 33.197 | 0.537 |
| Other Race - Including Multi-Racial | 6.605 | -20.445, 33.654 | 0.627 |
| **Education** |  |  |  |
| below high school | — | — |  |
| college or above | 0.112 | -16.125, 16.348 | 0.989 |
| high school | 3.530 | -13.881, 20.941 | 0.687 |
| **PIR** |  |  |  |
| <1 | — | — |  |
| 1–1.99 | -15.461 | -40.571, 9.649 | 0.223 |
| 2–3.99 | -24.919 | -47.904, -1.934 | 0.034 |
| ≥4 | -32.085 | -56.355, -7.815 | 0.010 |
| **Marriage** |  |  |  |
| Married/living with partner | — | — |  |
| Never married | 0.436 | -8.990, 9.862 | 0.927 |
| Widowed/divorced | 10.164 | -5.784, 26.111 | 0.207 |
| **Smoke** |  |  |  |
| Current smoker | — | — |  |
| Former smoker | 5.960 | -6.594, 18.515 | 0.346 |
| Never smoker | 6.426 | -9.416, 22.268 | 0.421 |
| **Drink** |  |  |  |
| Heavy drinker | — | — |  |
| Low to moderate drinker | -8.163 | -18.654, 2.328 | 0.125 |
| Nondrinker | -10.546 | -28.911, 7.819 | 0.256 |
| **BMI** |  |  |  |
| Low to normal (<25) | — | — |  |
| Obese (≥30) | -18.557 | -28.842, -8.272 | <0.001 |
| Overweight (25–30) | -10.914 | -20.873, -0.956 | 0.032 |
| **Cancer** |  |  |  |
| no | — | — |  |
| yes | 0.397 | -23.892, 24.686 | 0.974 |
| **Hypertension** |  |  |  |
| no | — | — |  |
| yes | 36.571 | 19.772, 53.369 | <0.001 |
| **Diabetes** |  |  |  |
| yes | — | — |  |
| no | -92.176 | -128.832, -55.519 | <0.001 |
| **Hyperuricaemia** |  |  |  |
| no | — | — |  |
| yes | 26.787 | 11.951, 41.623 | <0.001 |
| **Anemia** |  |  |  |
| no | — | — |  |
| yes | 37.265 | -7.168, 81.699 | 0.099 |
| **P** | 21.987 | 2.147, 41.827 | 0.030 |
| **Ca** | -24.899 | -67.058, 17.260 | 0.242 |
| **CLR** | 12.392 | 1.272, 23.513 | 0.030 |

Multivariable model 3 was adjusted for age, sex, Race/ethnicity, Marital status, Education, PIR, Smoke, BMI, Drink, Hypertension, Cancer, Diabetes, Anemia, Hyperuricaemia, P and Ca. BMI, body mass index; CLR, C-reactive protein to lymphocyte ratio; PIR, poverty income ratio; UACR, urinary albumin-to-creatinine ratio; 95% CI, 95% confidence interval.

**Supplementary Table S8 Weighted sensitivity analyses for incidence of CLR and CKD**

| **Characteristic** | **OR** | **95% CI** | **p-value** |
| --- | --- | --- | --- |
| **Age** |  |  |  |
| ≥65 | — | — |  |
| 20–44 | 4.64 | 3.78, 5.70 | <0.001 |
| 45–64 | 3.08 | 2.61, 3.64 | <0.001 |
| **Sex** |  |  |  |
| female | — | — |  |
| male | 1.04 | 0.91, 1.19 | 0.544 |
| **Race** |  |  |  |
| Mexican American | — | — |  |
| Other Hispanic | 1.28 | 0.94, 1.73 | 0.113 |
| Non-Hispanic White | 1.33 | 1.13, 1.56 | 0.001 |
| Non-Hispanic Black | 0.99 | 0.83, 1.19 | 0.939 |
| Other Race - Including Multi-Racial | 1.12 | 0.79, 1.59 | 0.509 |
| **Education** |  |  |  |
| below high school | — | — |  |
| college or above | 1.19 | 0.97, 1.46 | 0.089 |
| high school | 1.07 | 0.90, 1.28 | 0.431 |
| **PIR** |  |  |  |
| <1 | — | — |  |
| 1–1.99 | 1.02 | 0.82, 1.26 | 0.870 |
| 2–3.99 | 1.13 | 0.89, 1.43 | 0.299 |
| ≥4 | 1.30 | 1.03, 1.65 | 0.026 |
| **Marriage** |  |  |  |
| Married/living with partner | — | — |  |
| Never married | 0.84 | 0.65, 1.07 | 0.148 |
| Widowed/divorced | 0.72 | 0.61, 0.85 | <0.001 |
| **Smoke** |  |  |  |
| Current smoker | — | — |  |
| Former smoker | 0.90 | 0.75, 1.09 | 0.281 |
| Never smoker | 1.08 | 0.88, 1.32 | 0.456 |
| **Drinke** |  |  |  |
| Heavy drinker | — | — |  |
| Low to moderate drinker | 1.09 | 0.91, 1.30 | 0.357 |
| Nondrinker | 1.03 | 0.81, 1.30 | 0.815 |
| **BMI** |  |  |  |
| Low to normal (<25) | — | — |  |
| Obese (≥30) | 1.20 | 0.98, 1.47 | 0.079 |
| Overweight (25–30) | 1.33 | 1.10, 1.60 | 0.003 |
| **Cancer** |  |  |  |
| no | — | — |  |
| yes | 0.88 | 0.70, 1.11 | 0.289 |
| **Hypertension** |  |  |  |
| no | — | — |  |
| yes | 0.59 | 0.50, 0.68 | <0.001 |
| **Diabetes** |  |  |  |
| yes | — | — |  |
| no | 2.55 | 2.08, 3.13 | <0.001 |
| **Hyperuricaemia** |  |  |  |
| no | — | — |  |
| yes | 0.54 | 0.46, 0.64 | <0.001 |
| **Anemia** |  |  |  |
| no | — | — |  |
| yes | 0.52 | 0.41, 0.65 | <0.001 |
| **P** | 0.90 | 0.77, 1.05 | 0.175 |
| **Ca** | 0.96 | 0.78, 1.19 | 0.718 |
| **Stroke** |  |  |  |
| no | — | — |  |
| yes | 0.61 | 0.44, 0.84 | 0.003 |
| **Heart** |  |  |  |
| no | — | — |  |
| yes | 0.63 | 0.50, 0.79 | <0.001 |
| **CLR** | 0.78 | 0.70, 0.88 | <0.001 |

BMI, body mass index; CLR, C-reactive protein to lymphocyte ratio; CKD, chronic kidney disease; OR, odds ratio; PIR, poverty income ratio; 95% CI, 95% confidence interval.

**Supplementary Table S9 Weighted sensitivity analyses of CLR and eGFR levels**

| **Characteristic** | **Beta** | **95% CI** | **p-value** |
| --- | --- | --- | --- |
| **Age** |  |  |  |
| ≥65 | — | — |  |
| 20–44 | 30.456 | 29.270, 31.641 | <0.001 |
| 45–64 | 16.385 | 15.151, 17.619 | <0.001 |
| **Sex** |  |  |  |
| female | — | — |  |
| male | -0.908 | -1.729, -0.087 | 0.031 |
| **Race** |  |  |  |
| Mexican American | — | — |  |
| Other Hispanic | -3.361 | -5.735, -0.987 | 0.006 |
| Non-Hispanic White | -11.378 | -12.852, -9.904 | <0.001 |
| Non-Hispanic Black | -14.07 | -15.875, -12.266 | <0.001 |
| Other Race - Including Multi-Racial | -4.787 | -6.99, -2.584 | <0.001 |
| **Education** |  |  |  |
| below high school | — | — |  |
| college or above | 1.430 | 0.181, 2.679 | 0.026 |
| high school | 0.780 | -0.45, 2.009 | 0.210 |
| **PIR** |  |  |  |
| <1 | — | — |  |
| 1–1.99 | -3.088 | -4.435, -1.741 | <0.001 |
| 2–3.99 | -3.334 | -4.865, -1.803 | <0.001 |
| ≥4 | -4.302 | -5.720, -2.885 | <0.001 |
| **Marriage** |  |  |  |
| Married/living with partner | — | — |  |
| Never married | 6.918 | 5.841, 7.994 | <0.001 |
| Widowed/divorced | -7.529 | -8.615, -6.443 | <0.001 |
| **Smoke** |  |  |  |
| Current smoker | — | — |  |
| Former smoker | -4.187 | -5.383, -2.992 | <0.001 |
| Never smoker | -6.036 | -7.330, -4.743 | <0.001 |
| **Drinke** |  |  |  |
| Heavy drinker | — | — |  |
| Low to moderate drinker | -3.403 | -4.335, -2.472 | <0.001 |
| Nondrinker | -3.262 | -4.906, -1.618 | <0.001 |
| **BMI** |  |  |  |
| Low to normal (<25) | — | — |  |
| Obese (≥30) | 1.621 | 0.677, 2.564 | 0.001 |
| Overweight (25–30) | -2.051 | -3.119, -0.982 | <0.001 |
| **Cancer** |  |  |  |
| no | — | — |  |
| yes | -8.448 | -10.039, -6.857 | <0.001 |
| **Hypertension** |  |  |  |
| no | — | — |  |
| yes | -7.984 | -8.936, -7.033 | <0.001 |
| **Diabetes** |  |  |  |
| yes | — | — |  |
| no | 3.134 | 1.827, 4.441 | <0.001 |
| **Hyperuricaemia** |  |  |  |
| no | — | — |  |
| yes | -7.004 | -8.300, -5.708 | <0.001 |
| **Anemia** |  |  |  |
| no | — | — |  |
| yes | -4.399 | -6.187, -2.611 | <0.001 |
| **P** | -2.939 | -3.830, -2.048 | <0.001 |
| **Ca** | -3.798 | -5.364, -2.231 | <0.001 |
| **Stroke** |  |  |  |
| no | — | — |  |
| yes | -11.404 | -14.048, -8.760 | <0.001 |
| **Heart** |  |  |  |
| no | — | — |  |
| yes | -9.675 | -11.642, -7.709 | <0.001 |
| **CLR** | -0.978 | -1.848, -0.108 | 0.028 |

BMI, body mass index; CLR, C-reactive protein to lymphocyte ratio; eGFR: estimated glomerular filtration rate; PIR, poverty income ratio; 95% CI, 95% confidence interval.

**Supplementary Table S10 Weighted sensitivity analyses of CLR and UACR levels**

| **Characteristic** | **Beta** | **95% CI** | **p-value** |
| --- | --- | --- | --- |
| **Age** |  |  |  |
| ≥65 | — | — |  |
| 20–44 | 4.453 | -14.279, 23.184 | 0.636 |
| 45–64 | 17.512 | -4.802, 39.825 | 0.122 |
| **Sex** |  |  |  |
| female | — | — |  |
| male | 8.852 | -4.282, 21.986 | 0.183 |
| **Race** |  |  |  |
| Mexican American | — | — |  |
| Other Hispanic | 0.072 | -29.057, 29.202 | 0.996 |
| Non-Hispanic White | -11.719 | -25.793, 2.355 | 0.101 |
| Non-Hispanic Black | 6.943 | -18.497, 32.382 | 0.587 |
| Other Race - Including Multi-Racial | 4.390 | -22.997, 31.777 | 0.750 |
| **Education** |  |  |  |
| below high school | — | — |  |
| college or above | 1.294 | -14.928, 17.515 | 0.874 |
| high school | 4.919 | -12.408, 22.245 | 0.572 |
| **PIR** |  |  |  |
| <1 | — | — |  |
| 1–1.99 | -14.318 | -39.78, 11.145 | 0.265 |
| 2–3.99 | -22.796 | -45.858, 0.267 | 0.053 |
| ≥4 | -29.457 | -53.871, -5.044 | 0.019 |
| **Marriage** |  |  |  |
| Married/living with partner | — | — |  |
| Never married | 1.102 | -8.415, 10.619 | 0.818 |
| Widowed/divorced | 9.856 | -5.914, 25.627 | 0.216 |
| **Smoke** |  |  |  |
| Current smoker | — | — |  |
| Former smoker | 7.168 | -5.677, 20.013 | 0.269 |
| Never smoker | 6.472 | -9.276, 22.221 | 0.414 |
| **Drinke** |  |  |  |
| Heavy drinker | — | — |  |
| Low to moderate drinker | -8.098 | -18.803, 2.607 | 0.136 |
| Nondrinker | -10.085 | -28.381, 8.211 | 0.275 |
| **BMI** |  |  |  |
| Low to normal (<25) | — | — |  |
| Obese (≥30) | -18.501 | -28.751, -8.251 | <0.001 |
| Overweight (25–30) | -10.896 | -20.776, -1.016 | 0.031 |
| **Cancer** |  |  |  |
| no | — | — |  |
| yes | -0.932 | -25.282, 23.419 | 0.939 |
| **Hypertension** |  |  |  |
| no | — | — |  |
| yes | 32.581 | 16.650, 48.511 | <0.001 |
| **Diabetes** |  |  |  |
| yes | — | — |  |
| no | -88.326 | -125.3, -51.353 | <0.001 |
| **Hyperuricaemia** |  |  |  |
| no | — | — |  |
| yes | 25.743 | 10.908, 40.579 | <0.001 |
| **Anemia** |  |  |  |
| no | — | — |  |
| yes | 34.538 | -8.396, 77.471 | 0.113 |
| **P** | 21.289 | 1.781, 40.797 | 0.033 |
| Ca | -24.072 | -66.077, 17.934 | 0.256 |
| **Stroke** |  |  |  |
| no | — | — |  |
| yes | 38.823 | -16.233, 93.878 | 0.164 |
| **Heart** |  |  |  |
| no | — | — |  |
| yes | 42.078 | -7.950, 92.106 | 0.098 |
| **CLR** | 11.768 | 0.830, 22.706 | 0.035 |

BMI, body mass index; CLR, C-reactive protein to lymphocyte ratio; PIR, poverty income ratio; UACR, urinary albumin-to-creatinine ratio; 95% CI, 95% confidence interval.

**Supplementary Table S11 Unweighted Sensitivity Analyses of CLR and CKD Incidence Rates**

| **Characteristic** | **OR** | **95% CI** | **p-value** |
| --- | --- | --- | --- |
| **Age** |  |  |  |
| ≥65 | — | — |  |
| 20–44 | 5.10 | 4.37, 5.97 | <0.001 |
| 45–64 | 3.14 | 2.78, 3.56 | <0.001 |
| **Sex** |  |  |  |
| female | — | — |  |
| male | 0.93 | 0.83, 1.03 | 0.178 |
| **Race** |  |  |  |
| Mexican American | — | — |  |
| Other Hispanic | 1.29 | 1.02, 1.64 | 0.035 |
| Non-Hispanic White | 1.06 | 0.92, 1.23 | 0.410 |
| Non-Hispanic Black | 0.88 | 0.74, 1.04 | 0.137 |
| Other Race - Including Multi-Racial | 0.94 | 0.71, 1.27 | 0.699 |
| **Education** |  |  |  |
| below high school | — | — |  |
| college or above | 1.15 | 1.01, 1.31 | 0.040 |
| high school | 1.07 | 0.93, 1.23 | 0.329 |
| **PIR** |  |  |  |
| <1 | — | — |  |
| 1–1.99 | 1.09 | 0.94, 1.26 | 0.259 |
| 2–3.99 | 1.21 | 1.04, 1.41 | 0.015 |
| ≥4 | 1.40 | 1.18, 1.67 | <0.001 |
| **Marriage** |  |  |  |
| Married/living with partner | — | — |  |
| Never married | 0.91 | 0.76, 1.08 | 0.276 |
| Widowed/divorced | 0.76 | 0.67, 0.85 | <0.001 |
| **Smoke** |  |  |  |
| Current smoker | — | — |  |
| Former smoker | 0.95 | 0.82, 1.10 | 0.506 |
| Never smoker | 1.04 | 0.89, 1.21 | 0.642 |
| **Drinke** |  |  |  |
| Heavy drinker | — | — |  |
| Low to moderate drinker | 1.09 | 0.97, 1.22 | 0.168 |
| Nondrinker | 1.03 | 0.89, 1.20 | 0.683 |
| **BMI** |  |  |  |
| Low to normal (<25) | — | — |  |
| Obese (≥30) | 1.20 | 1.05, 1.37 | 0.009 |
| Overweight (25–30) | 1.30 | 1.14, 1.48 | <0.001 |
| **Cancer** |  |  |  |
| no | — | — |  |
| yes | 0.83 | 0.71, 0.96 | 0.015 |
| **Hypertension** |  |  |  |
| no | — | — |  |
| yes | 0.54 | 0.49, 0.61 | <0.001 |
| **Diabetes** |  |  |  |
| yes | — | — |  |
| no | 2.65 | 2.35, 2.98 | <0.001 |
| **Hyperuricaemia** |  |  |  |
| no | — | — |  |
| yes | 0.52 | 0.46, 0.58 | <0.001 |
| **Anemia** |  |  |  |
| no | — | — |  |
| yes | 0.49 | 0.41, 0.57 | <0.001 |
| **P** | 0.92 | 0.84, 1.02 | 0.099 |
| **Ca** | 0.97 | 0.85, 1.11 | 0.660 |
| **CLR** | 0.81 | 0.75, 0.87 | <0.001 |

BMI, body mass index; CLR, C-reactive protein to lymphocyte ratio; CKD, chronic kidney disease; OR, odds ratio; PIR, poverty income ratio; 95% CI, 95% confidence interval.

**Supplementary Table S12 Unweighted Sensitivity Analyses of CLR and eGFR Levels**

| **Characteristic** | **Beta** | **95% CI** | **p-value** |
| --- | --- | --- | --- |
| **Age** |  |  |  |
| ≥65 | — | — |  |
| 20–44 | 32.914 | 32.067, 33.762 | <0.001 |
| 45–64 | 17.079 | 16.306, 17.851 | <0.001 |
| **Sex** |  |  |  |
| female | — | — |  |
| male | -4.349 | -5.046, -3.652 | <0.001 |
| **Race** |  |  |  |
| Mexican American | — | — |  |
| Other Hispanic | -2.990 | -4.395, -1.586 | <0.001 |
| Non-Hispanic White | -11.314 | -12.241, -10.387 | <0.001 |
| Non-Hispanic Black | -12.739 | -13.816, -11.661 | <0.001 |
| Other Race - Including Multi-Racial | -4.116 | -5.886, -2.346 | <0.001 |
| **Education** |  |  |  |
| below high school | — | — |  |
| college or above | 2.672 | 1.802, 3.543 | <0.001 |
| high school | 2.207 | 1.287, 3.127 | <0.001 |
| **PIR** |  |  |  |
| <1 | — | — |  |
| 1–1.99 | -2.824 | -3.782, -1.865 | <0.001 |
| 2–3.99 | -3.182 | -4.179, -2.186 | <0.001 |
| ≥4 | -3.205 | -4.287, -2.123 | <0.001 |
| **Marriage** |  |  |  |
| Married/living with partner | — | — |  |
| Never married | 6.720 | 5.774, 7.666 | <0.001 |
| Widowed/divorced | -9.065 | -9.893, -8.236 | <0.001 |
| **Smoke** |  |  |  |
| Current smoker | — | — |  |
| Former smoker | -4.027 | -4.900, -3.155 | <0.001 |
| Never smoker | -7.253 | -8.217, -6.289 | <0.001 |
| **Drinke** |  |  |  |
| Heavy drinker | — | — |  |
| Low to moderate drinker | -3.230 | -3.975, -2.485 | <0.001 |
| Nondrinker | -3.612 | -4.635, -2.590 | <0.001 |
| **BMI** |  |  |  |
| Low to normal (<25) | — | — |  |
| Obese (≥30) | 3.275 | 2.418, 4.132 | <0.001 |
| Overweight (25–30) | -0.502 | -1.310, 0.307 | 0.220 |
| **Cancer** |  |  |  |
| no | — | — |  |
| yes | -9.153 | -10.303, -8.004 | <0.001 |
| **Hypertension** |  |  |  |
| no | — | — |  |
| yes | -11.152 | -11.893, -10.411 | <0.001 |
| **Diabetes** |  |  |  |
| yes | — | — |  |
| no | 6.433 | 5.495, 7.372 | <0.001 |
| **Hyperuricaemia** |  |  |  |
| no | — | — |  |
| yes | -9.784 | -10.616, -8.952 | <0.001 |
| **Anemia** |  |  |  |
| no | — | — |  |
| yes | -5.422 | -6.630, -4.213 | <0.001 |
| **P** | -1.703 | -2.312, -1.093 | <0.001 |
| **Ca** | -4.682 | -5.583, -3.781 | <0.001 |
| **CLR** | -1.144 | -1.666, -0.623 | <0.001 |

BMI, body mass index; CLR, C-reactive protein to lymphocyte ratio; eGFR: estimated glomerular filtration rate; PIR, poverty income ratio; 95% CI, 95% confidence interval.

**Supplementary Table S13 Unweighted Sensitivity Analysis of CLR and UACR Levels**

| **Characteristic** | **Beta** | **95% CI** | **p-value** |
| --- | --- | --- | --- |
| **Age** |  |  |  |
| ≥65 | — | — |  |
| 20–44 | -9.995 | -32.75, 12.759 | 0.4 |
| 45–64 | -5.053 | -25.783, 15.678 | 0.6 |
| **Sex** |  |  |  |
| female | — | — |  |
| male | 26.540 | 10.836, 42.244 | <0.001 |
| **Race** |  |  |  |
| Mexican American | — | — |  |
| Other Hispanic | -14.249 | -45.888, 17.39 | 0.4 |
| Non-Hispanic White | -19.865 | -40.891, 1.16 | 0.064 |
| Non-Hispanic Black | -3.182 | -27.443, 21.079 | 0.8 |
| Other Race - Including Multi-Racial | 1.447 | -38.412, 41.307 | >0.9 |
| **Education** |  |  |  |
| below high school | — | — |  |
| college or above | -11.162 | -30.974, 8.650 | 0.3 |
| high school | -11.072 | -31.903, 9.759 | 0.3 |
| **PIR** |  |  |  |
| <1 | — | — |  |
| 1–1.99 | -38.847 | -60.47, -17.224 | <0.001 |
| 2–3.99 | -40.996 | -63.481, -18.510 | <0.001 |
| ≥4 | -49.443 | -73.977, -24.909 | <0.001 |
| **Marriage** |  |  |  |
| Married/living with partner | — | — |  |
| Never married | -5.874 | -27.623, 15.875 | 0.6 |
| Widowed/divorced | 7.218 | -11.859, 26.295 | 0.5 |
| **Smoke** |  |  |  |
| Current smoker | — | — |  |
| Former smoker | 10.670 | -9.146, 30.486 | 0.3 |
| Never smoker | 5.378 | -16.823, 27.578 | 0.6 |
| **Drinke** |  |  |  |
| Heavy drinker | — | — |  |
| Low to moderate drinker | -8.376 | -25.231, 8.479 | 0.3 |
| Nondrinker | -21.641 | -44.775, 1.494 | 0.067 |
| **BMI** |  |  |  |
| Low to normal (<25) | — | — |  |
| Obese (≥30) | -38.806 | -58.232, -19.38 | <0.001 |
| Overweight (25–30) | -21.792 | -40.009, -3.574 | 0.019 |
| **Cancer** |  |  |  |
| no | — | — |  |
| yes | -10.323 | -36.706, 16.060 | 0.4 |
| **Hypertension** |  |  |  |
| no | — | — |  |
| yes | 58.368 | 40.988, 75.747 | <0.001 |
| **Diabetes** |  |  |  |
| yes | — | — |  |
| no | -143.222 | -164.693, -121.751 | <0.001 |
| **Hyperuricaemia** |  |  |  |
| no | — | — |  |
| yes | 42.046 | 23.267, 60.825 | <0.001 |
| **Anemia** |  |  |  |
| no | — | — |  |
| yes | 61.096 | 33.824, 88.368 | <0.001 |
| **P** | 25.402 | 11.659, 39.146 | <0.001 |
| **Ca** | -66.129 | -86.44, -45.819 | <0.001 |
| **CLR** | 11.744 | -0.019, 23.506 | 0.050 |

BMI, body mass index; CLR, C-reactive protein to lymphocyte ratio; PIR, poverty income ratio; UACR, urinary albumin-to-creatinine ratio; 95% CI, 95% confidence interval.

**Supplementary Table S14 Sensitivity Analyses of CLR and CKD Incidence Rates (eGFR** **<45 ml/min/1.73m² or ACR ≥30 mg/g)**

| **Characteristic** | **OR** | **95% CI** | **p-value** |
| --- | --- | --- | --- |
| **Age** |  |  |  |
| ≥65 | — | — |  |
| 20–44 | 3.25 | 2.63, 4.01 | <0.001 |
| 45–64 | 2.36 | 1.98, 2.81 | <0.001 |
| **Sex** |  |  |  |
| female | — | — |  |
| male | 0.97 | 0.84, 1.11 | 0.615 |
| **Race** |  |  |  |
| Mexican American | — | — |  |
| Other Hispanic | 1.24 | 0.92, 1.67 | 0.163 |
| Non-Hispanic White | 1.37 | 1.15, 1.63 | <0.001 |
| Non-Hispanic Black | 1.13 | 0.94, 1.37 | 0.191 |
| Other Race - Including Multi-Racial | 0.99 | 0.72, 1.36 | 0.964 |
| **Education** |  |  |  |
| below high school | — | — |  |
| college or above | 1.28 | 1.04, 1.59 | 0.023 |
| high school | 1.18 | 0.98, 1.41 | 0.072 |
| **PIR** |  |  |  |
| <1 | — | — |  |
| 1–1.99 | 1.05 | 0.86, 1.28 | 0.615 |
| 2–3.99 | 1.17 | 0.94, 1.47 | 0.163 |
| ≥4 | 1.42 | 1.11, 1.81 | 0.006 |
| **Marriage** |  |  |  |
| Married/living with partner | — | — |  |
| Never married | 0.83 | 0.65, 1.06 | 0.137 |
| Widowed/divorced | 0.71 | 0.59, 0.85 | <0.001 |
| **Smoke** |  |  |  |
| Current smoker | — | — |  |
| Former smoker | 0.99 | 0.81, 1.21 | 0.928 |
| Never smoker | 1.14 | 0.93, 1.40 | 0.201 |
| **Drinke** |  |  |  |
| Heavy drinker | — | — |  |
| Low to moderate drinker | 1.18 | 0.98, 1.41 | 0.078 |
| Nondrinker | 1.03 | 0.81, 1.31 | 0.793 |
| **BMI** |  |  |  |
| Low to normal (<25) | — | — |  |
| Obese (≥30) | 1.16 | 0.94, 1.43 | 0.157 |
| Overweight (25–30) | 1.34 | 1.08, 1.65 | 0.008 |
| **Cancer** |  |  |  |
| no | — | — |  |
| yes | 0.90 | 0.69, 1.17 | 0.429 |
| **Hypertension** |  |  |  |
| no | — | — |  |
| yes | 0.58 | 0.50, 0.68 | <0.001 |
| **Diabetes** |  |  |  |
| yes | — | — |  |
| no | 2.92 | 2.45, 3.48 | <0.001 |
| **Hyperuricaemia** |  |  |  |
| no | — | — |  |
| yes | 0.64 | 0.53, 0.77 | <0.001 |
| **Anemia** |  |  |  |
| no | — | — |  |
| yes | 0.52 | 0.41, 0.65 | <0.001 |
| **P** | 0.89 | 0.76, 1.04 | 0.154 |
| **Ca** | 1.03 | 0.82, 1.28 | 0.815 |
| **CLR** | 0.79 | 0.70, 0.89 | <0.001 |

BMI, body mass index; CLR, C-reactive protein to lymphocyte ratio; CKD, chronic kidney disease; OR, odds ratio; PIR, poverty income ratio; 95% CI, 95% confidence interval.

**Supplementary Table S15 Sensitivity Analyses of CLR and eGFR Levels (eGFR** **<45 ml/min/1.73m² or ACR ≥30 mg/g)**

| **Characteristic** | **Beta** | **95% CI** | **p-value** |
| --- | --- | --- | --- |
| **Age** |  |  |  |
| ≥65 | — | — |  |
| 20–44 | 31.356 | 30.219, 32.493 | <0.001 |
| 45–64 | 17.103 | 15.911, 18.295 | <0.001 |
| **Sex** |  |  |  |
| female | — | — |  |
| male | -1.535 | -2.275, -0.794 | <0.001 |
| **Race** |  |  |  |
| Mexican American | — | — |  |
| Other Hispanic | -1.940 | -4.084, 0.204 | 0.075 |
| Non-Hispanic White | -7.962 | -9.233, -6.691 | <0.001 |
| Non-Hispanic Black | -12.845 | -14.395, -11.296 | <0.001 |
| Other Race - Including Multi-Racial | -3.455 | -5.498, -1.412 | 0.001 |
| **Education** |  |  |  |
| below high school | — | — |  |
| college or above | -1.296 | -2.266, -0.319 | 0.010 |
| high school | -0.709 | -1.801, 0.382 | 0.2 |
| **PIR** |  |  |  |
| <1 | — | — |  |
| 1–1.99 | -1.261 | -2.417, -0.104 | 0.033 |
| 2–3.99 | -1.350 | -2.708, 0.010 | 0.052 |
| ≥4 | -1.940 | -3.264, -0.614 | 0.005 |
| **Marriage** |  |  |  |
| Married/living with partner | — | — |  |
| Never married | 2.851 | 1.781, 3.920 | <0.001 |
| Widowed/divorced | -2.975 | -3.922, -2.027 | <0.001 |
| **Smoke** |  |  |  |
| Current smoker | — | — |  |
| Former smoker | -2.268 | -3.290, -1.247 | <0.001 |
| Never smoker | -1.136 | -2.130, -0.142 | 0.026 |
| **Drinke** |  |  |  |
| Heavy drinker | — | — |  |
| Low to moderate drinker | -1.074 | -1.903, -0.246 | 0.012 |
| Nondrinker | -0.036 | -1.315, 1.244 | >0.9 |
| **BMI** |  |  |  |
| Low to normal (<25) | — | — |  |
| Obese (≥30) | 0.296 | -0.480, 1.072 | 0.4 |
| Overweight (25–30) | -1.415 | -2.335, -0.495 | 0.003 |
| **Cancer** |  |  |  |
| no | — | — |  |
| yes | -2.115 | -3.418, -0.811 | 0.002 |
| **Hypertension** |  |  |  |
| no | — | — |  |
| yes | -3.578 | -4.413, -2.743 | <0.001 |
| **Diabetes** |  |  |  |
| yes | — | — |  |
| no | -0.780 | -1.846, 0.285 | 0.15 |
| **Hyperuricaemia** |  |  |  |
| no | — | — |  |
| yes | -6.260 | -7.318, -5.202 | <0.001 |
| **Anemia** |  |  |  |
| no | — | — |  |
| yes | -3.160 | -4.804, -1.516 | <0.001 |
| **P** | -3.295 | -4.171, -2.418 | <0.001 |
| **Ca** | -2.538 | -3.835, -1.241 | <0.001 |
| **CLR** | -1.054 | -1.961, -0.147 | 0.024 |

BMI, body mass index; CLR, C-reactive protein to lymphocyte ratio; eGFR: estimated glomerular filtration rate; PIR, poverty income ratio; 95% CI, 95% confidence interval.

**Supplementary Table S16 Sensitivity Analyses of CLR and UACR Levels (eGFR** **<45 ml/min/1.73m² or ACR ≥30 mg/g)**

| **Characteristic** | **Beta** | **95% CI** | **p-value** |
| --- | --- | --- | --- |
| **Age** |  |  |  |
| ≥65 | — | — |  |
| 20–44 | 0.998 | -16.361, 18.358 | 0.909 |
| 45–64 | 13.518 | -7.04, 34.075 | 0.194 |
| **Sex** |  |  |  |
| female | — | — |  |
| male | 9.196 | -1.978, 20.369 | 0.105 |
| **Race** |  |  |  |
| Mexican American | — | — |  |
| Other Hispanic | -0.322 | -29.227, 28.582 | 0.982 |
| Non-Hispanic White | -12.951 | -27.166, 1.264 | 0.073 |
| Non-Hispanic Black | 6.101 | -21.294, 33.496 | 0.658 |
| Other Race - Including Multi-Racial | 3.354 | -24.163, 30.87 | 0.808 |
| **Education** |  |  |  |
| below high school | — | — |  |
| college or above | 1.425 | -15.196, 18.046 | 0.865 |
| high school | 4.756 | -12.427, 21.938 | 0.582 |
| **PIR** |  |  |  |
| <1 | — | — |  |
| 1–1.99 | -11.857 | -36.757, 13.043 | 0.345 |
| 2–3.99 | -23.076 | -47.509, 1.358 | 0.064 |
| ≥4 | -29.371 | -53.953, -4.788 | 0.020 |
| **Marriage** |  |  |  |
| Married/living with partner | — | — |  |
| Never married | -0.563 | -10.398, 9.271 | 0.909 |
| Widowed/divorced | 8.148 | -7.895, 24.191 | 0.314 |
| **Smoke** |  |  |  |
| Current smoker | — | — |  |
| Former smoker | 3.897 | -9.351, 17.144 | 0.559 |
| Never smoker | 6.652 | -9.709, 23.014 | 0.420 |
| **Drinke** |  |  |  |
| Heavy drinker | — | — |  |
| Low to moderate drinker | -6.633 | -17.408, 4.142 | 0.223 |
| Nondrinker | -8.861 | -27.562, 9.840 | 0.347 |
| **BMI** |  |  |  |
| Low to normal (<25) | — | — |  |
| Obese (≥30) | -14.648 | -25.667, -3.628 | 0.010 |
| Overweight (25–30) | -8.287 | -19.647, 3.073 | 0.150 |
| **Cancer** |  |  |  |
| no | — | — |  |
| yes | 1.134 | -23.119, 25.386 | 0.926 |
| **Hypertension** |  |  |  |
| no | — | — |  |
| yes | 38.352 | 22.144, 54.560 | <0.001 |
| **Diabetes** |  |  |  |
| yes | — | — |  |
| no | -91.882 | -129.007, -54.757 | <0.001 |
| **Hyperuricaemia** |  |  |  |
| no | — | — |  |
| yes | 22.666 | 7.287, 38.046 | 0.005 |
| **Anemia** |  |  |  |
| no | — | — |  |
| yes | 39.084 | -9.319, 87.487 | 0.112 |
| **P** | 26.632 | 7.535, 45.729 | 0.007 |
| **Ca** | -27.294 | -0.801, 22.104 | 0.213 |
| **CLR** | 11.745 | 0.776, 22.714 | 0.036 |

BMI, body mass index; CLR, C-reactive protein to lymphocyte ratio; PIR, poverty income ratio; UACR, urinary albumin-to-creatinine ratio; 95% CI, 95% confidence interval.

**Supplementary Table S17 Sensitivity Analyses of CLR and CKD Incidence Rates Adjusted for Prescription Medications**

| **Characteristic** | **OR** | **95% CI** | **p-value** |
| --- | --- | --- | --- |
| **Age** |  |  |  |
| ≥65 | — | — |  |
| 20–44 | 4.48 | 3.64, 5.50 | <0.001 |
| 45–64 | 3.08 | 2.62, 3.63 | <0.001 |
| **Sex** |  |  |  |
| female | — | — |  |
| male | 1.01 | 0.88, 1.16 | 0.898 |
| **Race** |  |  |  |
| Mexican American | — | — |  |
| Other Hispanic | 1.26 | 0.93, 1.71 | 0.134 |
| Non-Hispanic White | 1.30 | 1.11, 1.53 | 0.002 |
| Non-Hispanic Black | 0.97 | 0.81, 1.17 | 0.775 |
| Other Race - Including Multi-Racial | 1.09 | 0.77, 1.53 | 0.621 |
| **Education** |  |  |  |
| below high school | — | — |  |
| college or above | 1.23 | 1.01, 1.51 | 0.044 |
| high school | 1.12 | 0.94, 1.34 | 0.196 |
| **PIR** |  |  |  |
| <1 | — | — |  |
| 1–1.99 | 1.07 | 0.88, 1.30 | 0.496 |
| 2–3.99 | 1.19 | 0.95, 1.50 | 0.129 |
| ≥4 | 1.39 | 1.10, 1.75 | 0.006 |
| **Marriage** |  |  |  |
| Married/living with partner | — | — |  |
| Never married | 0.85 | 0.67, 1.09 | 0.197 |
| Widowed/divorced | 0.70 | 0.59, 0.83 | <0.001 |
| **Smoke** |  |  |  |
| Current smoker | — | — |  |
| Former smoker | 0.93 | 0.78, 1.11 | 0.418 |
| Never smoker | 1.10 | 0.90, 1.35 | 0.326 |
| **Drinke** |  |  |  |
| Heavy drinker | — | — |  |
| Low to moderate drinker | 1.10 | 0.92, 1.32 | 0.293 |
| Nondrinker | 1.04 | 0.82, 1.33 | 0.716 |
| **BMI** |  |  |  |
| Low to normal (<25) | — | — |  |
| Obese (≥30) | 1.19 | 0.97, 1.46 | 0.087 |
| Overweight (25–30) | 1.35 | 1.13, 1.62 | 0.001 |
| **Cancer** |  |  |  |
| no | — | — |  |
| yes | 0.87 | 0.69, 1.10 | 0.238 |
| **Hypertension** |  |  |  |
| no | — | — |  |
| yes | 0.66 | 0.56, 0.77 | <0.001 |
| **Diabetes** |  |  |  |
| yes | — | — |  |
| no | 2.37 | 1.90, 2.96 | <0.001 |
| **Hyperuricaemia** |  |  |  |
| no | — | — |  |
| yes | 0.54 | 0.46, 0.64 | <0.001 |
| **Anemia** |  |  |  |
| no | — | — |  |
| yes | 0.51 | 0.41, 0.64 | <0.001 |
| **P** | 0.91 | 0.78, 1.06 | 0.209 |
| **Ca** | 0.98 | 0.80, 1.21 | 0.882 |
| **Lipid-lowering drugs** | 0.79 | 0.68, 0.93 | 0.005 |
| **Anti-inflammatory drugs** | 1.25 | 1.00, 1.58 | 0.051 |
| **Antidiabetic drugs** | 0.96 | 0.72, 1.28 | 0.781 |
| **Antihypertensive drugs** | 0.70 | 0.60, 0.83 | <0.001 |
| **CLR** | 0.78 | 0.69, 0.88 | <0.001 |

BMI, body mass index; CLR, C-reactive protein to lymphocyte ratio; CKD, chronic kidney disease; OR, odds ratio; PIR, poverty income ratio; 95% CI, 95% confidence interval.

**Supplementary Table S18 Sensitivity Analyses of CLR and eGFR Levels Adjusted for Prescription Medications**

| **Characteristic** | **Beta** | **95% CI** | **p-value** |
| --- | --- | --- | --- |
| **Age** |  |  |  |
| ≥65 | — | — |  |
| 20–44 | 29.843 | 28.673, 31.013 | <0.001 |
| 45–64 | 16.202 | 14.984, 17.420 | <0.001 |
| **Sex** |  |  |  |
| female | — | — |  |
| male | -0.977 | -1.776, -0.177 | 0.017 |
| **Race** |  |  |  |
| Mexican American | — | — |  |
| Other Hispanic | -3.361 | -5.726, -0.997 | 0.006 |
| Non-Hispanic White | -10.952 | -12.455, -9.450 | <0.001 |
| Non-Hispanic Black | -14.090 | -15.887, -12.294 | <0.001 |
| Other Race - Including Multi-Racial | -4.674 | -6.861, -2.486 | <0.001 |
| **Education** |  |  |  |
| below high school | — | — |  |
| college or above | 1.685 | 0.477, 2.892 | 0.007 |
| high school | 1.297 | 0.067, 2.526 | 0.039 |
| **PIR** |  |  |  |
| <1 | — | — |  |
| 1–1.99 | -2.802 | -4.175, -1.43 | <0.001 |
| 2–3.99 | -2.802 | -4.288, -1.316 | <0.001 |
| ≥4 | -3.534 | -5.003, -2.065 | <0.001 |
| **Marriage** |  |  |  |
| Married/living with partner | — | — |  |
| Never married | 6.636 | 5.554, 7.717 | <0.001 |
| Widowed/divorced | -7.397 | -8.54, -6.255 | <0.001 |
| **Smoke** |  |  |  |
| Current smoker | — | — |  |
| Former smoker | -3.674 | -4.793, -2.555 | <0.001 |
| Never smoker | -5.211 | -6.443, -3.980 | <0.001 |
| **Drinke** |  |  |  |
| Heavy drinker | — | — |  |
| Low to moderate drinker | -3.161 | -4.033, -2.289 | <0.001 |
| Nondrinker | -2.702 | -4.394, -1.010 | 0.002 |
| **BMI** |  |  |  |
| Low to normal (<25) | — | — |  |
| Obese (≥30) | 1.945 | 1.135, 2.756 | <0.001 |
| Overweight (25–30) | -1.595 | -2.58, -0.61 | 0.002 |
| **Cancer** |  |  |  |
| no | — | — |  |
| yes | -8.057 | -9.511, -6.604 | <0.001 |
| **Hypertension** |  |  |  |
| no | — | — |  |
| yes | -4.152 | -5.141, -3.163 | <0.001 |
| **Diabetes** |  |  |  |
| yes | — | — |  |
| no | 2.285 | 0.624, 3.947 | 0.008 |
| **Hyperuricaemia** |  |  |  |
| no | — | — |  |
| yes | -6.953 | -8.164, -5.741 | <0.001 |
| **Anemia** |  |  |  |
| no | — | — |  |
| yes | -4.331 | -6.136, -2.527 | <0.001 |
| **P** | -2.674 | -3.609, -1.739 | <0.001 |
| **Ca** | -3.550 | -5.054, -2.047 | <0.001 |
| **Lipid-lowering drugs** | -8.279 | -9.701, -6.858 | <0.001 |
| **Anti-inflammatory drugs** | -0.957 | -2.405, 0.491 | 0.191 |
| **Antidiabetic drugs** | 2.355 | -0.376, 5.086 | 0.090 |
| **Antihypertensive drugs** | -9.108 | -10.464, -7.752 | <0.001 |
| **CLR** | -1.101 | -1.943, -0.259 | 0.011 |

BMI, body mass index; CLR, C-reactive protein to lymphocyte ratio; eGFR: estimated glomerular filtration rate; PIR, poverty income ratio; 95% CI, 95% confidence interval.

**Supplementary Table S19 Sensitivity Analyses of CLR and UACR Levels Adjusted for Prescription Medications**

| **Characteristic** | **Beta** | **95% CI** | **p-value** |
| --- | --- | --- | --- |
| **Age** |  |  |  |
| ≥65 | — | — |  |
| 20–44 | 11.425 | -8.424, 31.275 | 0.254 |
| 45–64 | 23.057 | -1.054, 47.169 | 0.061 |
| **Sex** |  |  |  |
| female | — | — |  |
| male | 10.419 | -2.461, 23.299 | 0.110 |
| **Race** |  |  |  |
| Mexican American | — | — |  |
| Other Hispanic | 1.045 | -28.135, 30.226 | 0.943 |
| Non-Hispanic White | -12.170 | -25.947, 1.608 | 0.082 |
| Non-Hispanic Black | 10.557 | -18.523, 39.637 | 0.470 |
| Other Race - Including Multi-Racial | 4.1788 | -25.316, 33.673 | 0.778 |
| **Education** |  |  |  |
| below high school | — | — |  |
| college or above | -0.824 | -17.021, 15.372 | 0.919 |
| high school | 2.517 | -15.302, 20.335 | 0.778 |
| **PIR** |  |  |  |
| <1 | — | — |  |
| 1–1.99 | -18.255 | -42.932, 6.422 | 0.144 |
| 2–3.99 | -27.593 | -52.119, -3.067 | 0.028 |
| ≥4 | -33.259 | -59.332, -7.186 | 0.013 |
| **Marriage** |  |  |  |
| Married/living with partner | — | — |  |
| Never married | -0.021 | -9.66, 9.619 | 0.997 |
| Widowed/divorced | 12.552 | -2.446, 27.550 | 0.099 |
| **Smoke** |  |  |  |
| Current smoker | — | — |  |
| Former smoker | 4.968 | -7.424, 17.360 | 0.426 |
| Never smoker | 6.196 | -9.411, 21.804 | 0.430 |
| **Drinke** |  |  |  |
| Heavy drinker | — | — |  |
| Low to moderate drinker | -5.992 | -15.66, 3.677 | 0.220 |
| Nondrinker | -9.308 | -27.213, 8.598 | 0.303 |
| **BMI** |  |  |  |
| Low to normal (<25) | — | — |  |
| Obese (≥30) | -17.847 | -28.085, -7.609 | <0.001 |
| Overweight (25–30) | -15.225 | -23.460, -6.990 | <0.001 |
| **Cancer** |  |  |  |
| no | — | — |  |
| yes | -0.842 | -23.945, 22.262 | 0.942 |
| **Hypertension** |  |  |  |
| no | — | — |  |
| yes | 19.870 | 6.585, 33.155 | 0.004 |
| **Diabetes** |  |  |  |
| yes | — | — |  |
| no | -76.415 | -117.925, -34.904 | <0.001 |
| **Hyperuricaemia** |  |  |  |
| no | — | — |  |
| yes | 22.402 | 7.269, 37.535 | 0.004 |
| **Anemia** |  |  |  |
| no | — | — |  |
| yes | 36.816 | -6.992, 80.625 | 0.098 |
| **P** | 21.920 | 2.424, 41.416 | 0.028 |
| **Ca** | -26.251 | -68.451, 15.949 | 0.218 |
| **Lipid-lowering drugs** | 2.452 | -24.191, 29.095 | 0.855 |
| **Anti-inflammatory drugs** | -17.032 | -32.468, -1.597 | 0.031 |
| **Antidiabetic drugs** | 24.658 | -35.773, 85.088 | 0.418 |
| **Antihypertensive drugs** | 45.234 | 20.783, 69.684 | <0.001 |
| **CLR** | 10.220 | -1.362, 21.801 | 0.083 |

BMI, body mass index; CLR, C-reactive protein to lymphocyte ratio; PIR, poverty income ratio; UACR, urinary albumin-to-creatinine ratio; 95% CI, 95% confidence interval.

# Supplementary Figures


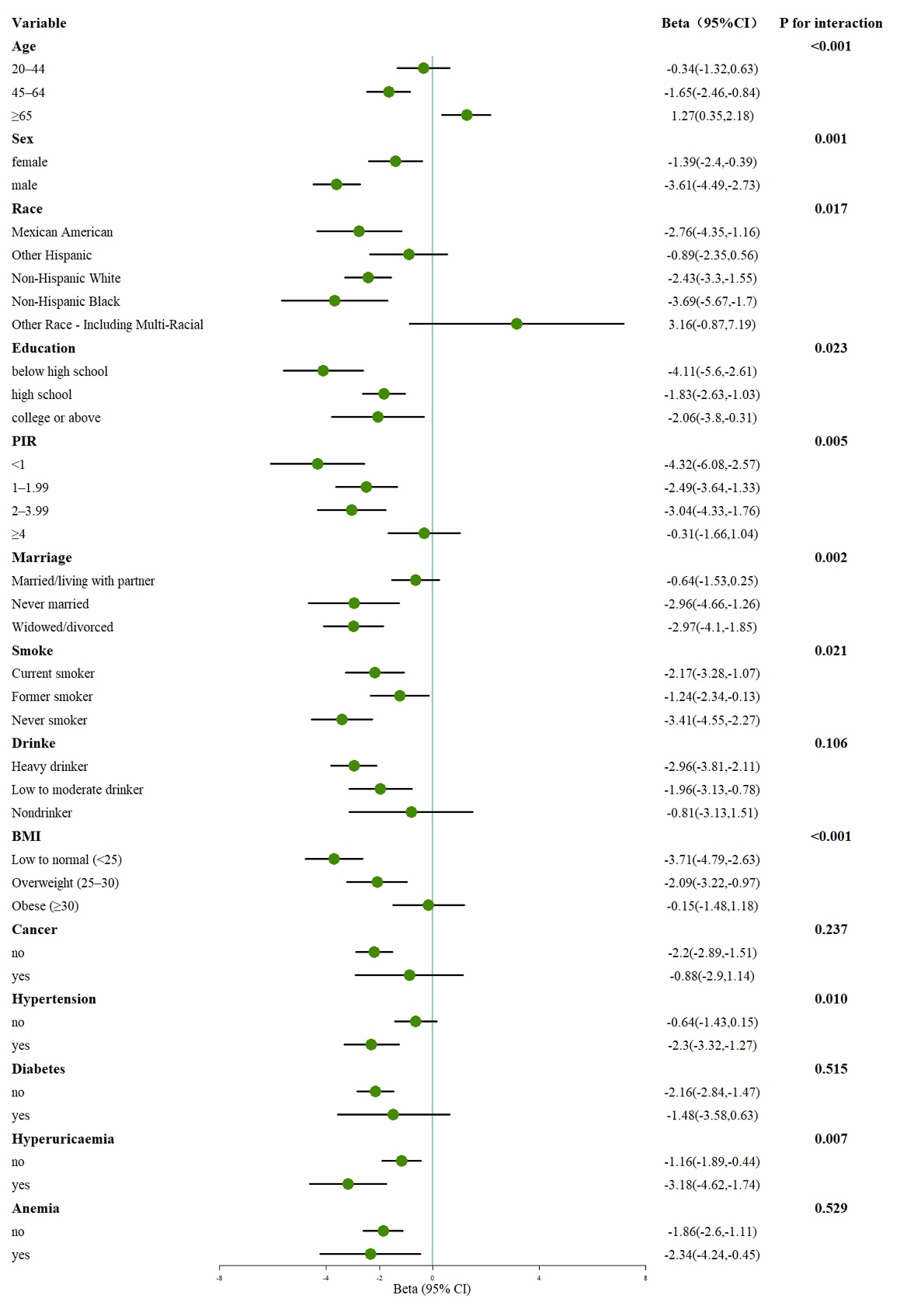


**Supplementary Figure S1 Subgroup analysis of CLR and eGFR levels**

BMI, body mass index; CLR, C-reactive protein to lymphocyte ratio; eGFR: estimated glomerular filtration rate; PIR, poverty income ratio; 95% CI, 95% confidence interval.


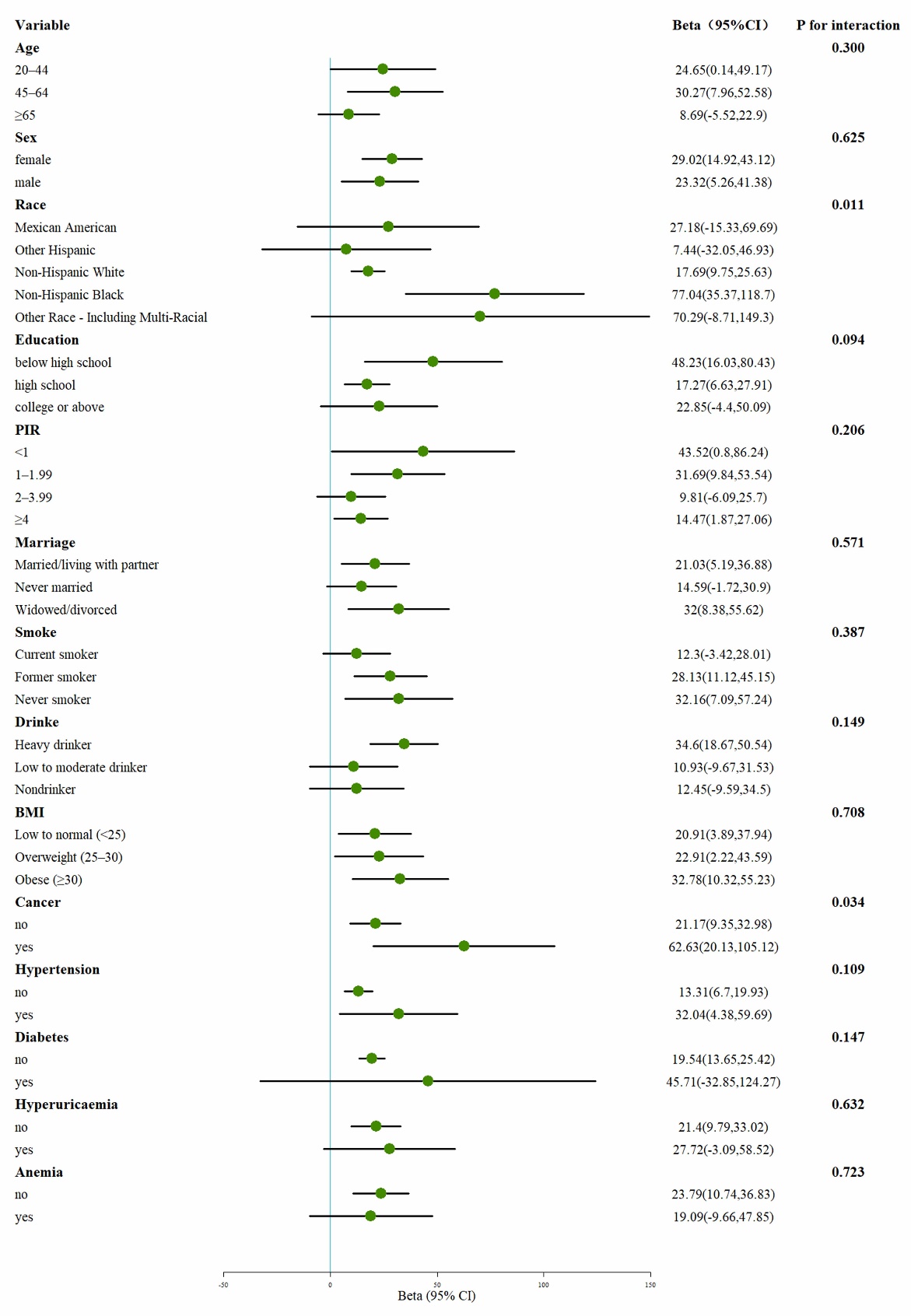


**Supplementary Figure S2 Subgroup analysis of CLR and UACR levels**

BMI, body mass index; CLR, C-reactive protein to lymphocyte ratio; PIR, poverty income ratio; UACR, urinary albumin-to-creatinine ratio; 95% CI, 95% confidence interval.
